# A short-oligonucleotide microarray that allows improved detection of gastrointestinal tract microbial communities

**DOI:** 10.1186/1471-2180-8-195

**Published:** 2008-11-11

**Authors:** Carl R Harrington, Sacha Lucchini, Karyn P Ridgway, Udo Wegmann, Tracy J Eaton, Jay CD Hinton, Michael J Gasson, Arjan Narbad

**Affiliations:** 1 Commensals and Microflora, Institute of Food Research, Norwich Research Park, Colney Lane, Norwich, Norfolk, NR4 7UA, UK; 2 Pathogens: Molecular Microbiology, Institute of Food Research, Norwich Research Park, Colney Lane, Norwich, Norfolk, NR4 7UA, UK

## Abstract

**Background:**

The human gastrointestinal (GI) tract contains a diverse collection of bacteria, most of which are unculturable by conventional microbiological methods. Increasingly molecular profiling techniques are being employed to examine this complex microbial community. The purpose of this study was to develop a microarray technique based on 16S ribosomal gene sequences for rapidly monitoring the microbial population of the GI tract.

**Results:**

We have developed a culture-independent, semi-quantitative, rapid method for detection of gut bacterial populations based on 16S rDNA probes using a DNA microarray. We compared the performance of microarrays based on long (40- and 50-mer) and short (16–21-mer) oligonucleotides. Short oligonucleotides consistently gave higher specificity. Optimal DNA amplification and labelling, hybridisation and washing conditions were determined using a probe with an increasing number of nucleotide mismatches, identifying the minimum number of nucleotides needed to distinguish between perfect and mismatch probes. An independent PCR-based control was used to normalise different hybridisation results, and to make comparisons between different samples, greatly improving the detection of changes in the gut bacterial population. The sensitivity of the microarray was determined to be 8.8 × 10^4 ^bacterial cells g^-1 ^faecal sample, which is more sensitive than a number of existing profiling methods. The short oligonucleotide microarray was used to compare the faecal flora from healthy individuals and a patient suffering from Ulcerative Colitis (UC) during the active and remission states. Differences were identified in the bacterial profiles between healthy individuals and a UC patient. These variations were verified by Denaturing Gradient Gel Electrophoresis (DGGE) and DNA sequencing.

**Conclusion:**

In this study we demonstrate the design, testing and application of a highly sensitive, short oligonucleotide community microarray. Our approach allows the rapid discrimination of bacteria inhabiting the human GI tract, at taxonomic levels ranging from species to the superkingdom bacteria. The optimised protocol is available at: . It offers a high throughput method for studying the dynamics of the bacterial population over time and between individuals.

## Background

The human gastrointestinal (GI) tract contains a complex community of bacteria with up to 1 × 10^12 ^bacteria per gram of luminal contents [1]. Currently, the function of GI tract bacteria in the maintenance of human health and in some disease states is generating intense interest. The microbiota is known to stimulate the immune system, produce vitamins and short chain fatty acids, help the digestive process and is involved in preventing colonisation by potentially pathogenic bacteria. It is estimated that there may be as many as 1000 different bacterial species within the human GI tract [2]. One of the main barriers to the progress of research in this area is the unculturable nature of many GI tract bacteria. However in the last decade, culture independent molecular profiling methods have been developed. Many of these methods are based on the 16S ribosomal gene which contains highly conserved nucleotides across all bacterial species, interspersed with regions of sequences which are variable. Such methods include Denaturing Gradient Gel Electrophoresis (DGGE) [3,4], Fluorescence *in situ *Hybridisation (FISH) [4,5] and cloning and sequencing of 16S ribosomal gene libraries [6]. While these methods have proven extremely valuable, they are limited by the number of samples that can be analysed and the time taken to process them.

DNA microarray technology is a potentially powerful tool for rapid high throughput detection of thousands of 16S ribosomal gene sequences and the provision of a profile of microbial communities. One of the first described uses of microarrays for detecting genes in an environmental sample was the construction of an oligonucleotide microchip to identify key bacteria of the nitrifying genera [7]. DNA microarrays have also been developed to identify selected bacteria from other environmental niches [8-11] and to detect bacterial strains from the human gut [12,13]. However the human gut microarray studies were only able to detect a limited number of species and did not provide information about the relative amounts of bacteria found in different individuals. A diversity microarray consisting of 40-mer probes has been developed, tested and optimised to detect a range of bacterial taxonomic hierarchies. This was used to study the adult human GI tract [14] and to monitor the development of the human infant intestinal microbiota [15].

Here we report the development and validation of a DNA based microarray that uses 230 shorter 16S ribosomal DNA gene-based probes, mainly 16–21-mer in length. Our approach allows the rapid detection of a large range of gut bacteria at various taxonomic hierarchies.

## Methods

### Bacterial strains

Reference strains used are listed in Table 1. These were obtained from the specified culture collections. Strains were propagated using the media recommended by the supplier.

**Table 1:** Seventeen reference strains used to test the specificity of microarrays

**Taxon**	**Source**^*a*^
*Bacteroides distasonis*	DSMZ 20701

*Bacteroides fragilis*	DSMZ 1396

*Bifidobacterium adolescentis*	DSMZ 20083

*Bifidobacterium angulatum*	DSMZ 20098

*Bifidobacterium bifidum*	DSMZ 20082

*Bifidobacterium infantis*	DSMZ 20088

*Bifidobacterium longum*	DSMZ 20219

*Bifidobacterium pseudocatenulatum*	DSMZ 20243

*Clostridium leptum*	DSMZ 753

*Enterococcus faecium*	CECT 410

*Escherichia coli*	CECT 434

*Lactobacillus acidophilus*	DSMZ 20079

*Lactobacillus gasseri*	DSMZ 20243

*Lactobacillus johnsonii*	FI 9785

*Thermus thermophilus*	DSMZ 579

*Ruminococcus obeum*	ATCC 29174

*Salmonella enterica *serovar Typhimurium (SL1344)	[52]

^*a *^DSMZ, Deutsche Sammlung von Mikroorganismen und Zellkulturen, Braunschweig, Germany; ATCC, American Type Culture Collection, Middlesex, UK; CECT, Spanish Type Culture Collection, Valencia, Spain; FI, Institute of Food Research Culture Collection, Norwich, UK.

### Genomic DNA extraction

Genomic DNA from pure bacterial cultures was isolated using a QIAGEN Genomic-tip 20/G (Qiagen Ltd., UK) following the manufacturer's instructions. Faecal samples from healthy volunteers or a patient suffering from Ulcerative Colitis were collected and stored frozen at -80°C within one hour of defecation. Faecal genomic DNA was extracted using the QIAamp DNA stool Mini Kit (Qiagen Ltd., UK) following the manufacturer's instructions. The patient with UC was taking prescribed medication during the study to reduce inflammation. This study was approved by the Medical Ethical Committee of the Norfolk and Norwich University Hospital and by the Suffolk Local Research Ethical Committee (Ref 06-Q0102-91).

### PCR amplification of 16S ribosomal genes

PCR products were amplified from either 5 ng faecal genomic DNA or 5 ng pure bacterial culture genomic DNA, using the Amp F and Amp R primers (Table 2). These primers generated a product of approximately 1.5 kb length from the genomic DNA templates. As an internal positive control, 10 pg of purified *Thermus thermophilus *genomic DNA was added to each 50 μl reaction. Reaction tubes contained 10 μl Phusion HF buffer (Finnzymes, Finland), 200 μM dNTPs (Bioline, UK), 0.5 μM each primer, 0.5 U of Phusion DNA polymerase (Finnzymes, Finland), 5 ng genomic DNA and deionised water (dH_2_O) to 50 μl. The initial DNA denaturation step was performed at 96°C for 4 min using a PCR sprint thermocycler (Hybaid, UK), followed by 15 cycles of 96°C for 1 min, 53°C for 1 min, and 72°C for 1 min 10 s. A final step of 72°C for 4 min completed the reaction. PCR products were purified using a Wizard PCR Purification Kit (Promega Corporation, WI, USA) and eluted with 50 μl of dH_2_O.

**Table 2:** 16S ribosomal gene primers used for PCR amplification or DGGE

**Primer**	**Sequence (5'-3')**	**Primer target site**^*a*^	**Reference**
Amp F	GAGAGTTTGATYCTGGCTCAG	0006	[13]

Amp R	AAGGAGGTGATCCARCCGCA	1530	[13]

InfL6^*b*^	TATCGGGGAGCAAGCGAG	0445	[53]

Bif662-r	CCACCGTTACACCGGGAA	0662	[3]

ENT183^*c*^	CGTCGCAAGACCAAAGAG	0183	[54]

424R	CCGCTGAAAGTACTTTACAACC	0424	This study

F-968^*d*^	GAACGCGAAGAACCTTAC	0968	[55]

R-1401	CGGTGTGTAGAAGACCC	1401	[55]

F-341^*d*^	CCTACGGGAGGCAGCAG	0341	[56]

R-534	ATTACCGCGGCTGCTGG	0534	[56]

^*a *^Primer target site according to *E. coli *16S ribosomal gene numbering as the reference.

^*b *^This primer was known as *B. longum *grp3 for the microarray.

^*c *^This primer was designated Enterobacteriaceae1 for the microarray.

^*d *^A 39 nt GC clamp was added to the 5' end of the primer when used for DGGE.

### PCR detection of Enterobacteriaceae and bifidobacteria

Enterobacteriaceae (ENT183 and 424R) and bifidobacteria (InfL6 and Bif662-r) primer sequences used to determine the comparative nature of the microarray are listed in Table 2. Reaction tubes contained 5 μl 10 × PCR buffer (Amersham Biosciences, UK), 200 μM dNTPs (Bioline, UK), 0.5 μM of either ENT183 and 424R or InfL6 and Bif662-r, 1 U of *Taq *DNA polymerase (Amersham Biosciences, UK), 5 ng faecal genomic DNA, and dH_2_O to 50 μl. PCR conditions for Enterobacteriaceae were: one cycle at 96°C for 4 min, followed by five step increments between 20 and 45 cycles of 96°C for 30 s, 56°C for 30 s, and 72°C for 30 s. PCR conditions for bifidobacteria were one cycle at 96°C for 4 min, followed by 5 cycle advancements between 20 and 45 cycles of 96°C for 35 s, 57°C for 30 s, and 72°C for 30 s. The analysis of gel images obtained after running the PCR products on 1.5% agarose gels was performed using the Phoretix V5.20 software (NonLinear Dynamics Limited, UK).

### DGGE analysis of faecal DNA

The variable V6-V8 region of 16S ribosomal gene was amplified from faecal DNA using primers F-968 and R-1401 and V3 region using F-341 and R-534 (Table 2). The reaction mixture contained 5 μl 10 × HotMaster buffer (Eppendorf, Germany), 200 μM dNTPs (Bioline, UK), 20 pmol each primer, 2.5 mM MgCl_2_, 1 U HotMaster polymerase (Eppendorf, Germany), 100 ng template DNA and dH_2_O to 50 μl. Reactions were carried out in a PCR machine (Hybaid, UK) with an initial denaturation cycle of 94°C for 2 min followed by 30 cycles of 94°C for 20 s, 58°C for 10 s, 65°C for 20 s with a final extension at 65°C for 5 min. PCR products were verified by gel electrophoresis for single band products. DGGE analysis was performed using the Ingeny PhorU-2 electrophoresis system according to manufacturer's instructions (Ingeny International BV, The Netherlands), using 8% polyacrylamide gels containing a gradient of 40–60% urea and formamide as denaturants. A solution of 100% denaturant is defined as 40% vol/vol formamide and 7.0 M urea. PCR amplicons were separated by electrophoresis at 80 V for 17 h and stained with SYBR Green (Molecular Probes) (1:10000 in 0.5 × TAE). Gels were viewed on a Dark Reader (Clare Chemicals, Colorado, USA) and photographed using DigiDoc software (Alpha Innotech, California, USA).

### Sequencing of DGGE bands

Bands of interest were excised and soaked in 50 μl dH_2_O to elute the DNA. Two microlitres of this sample was used as template to reamplify the DNA fragment. The PCR reaction used the same HotMaster polymerase procedure as described for the DGGE, except that the forward primer lacked a GC clamp. PCR products were purified using a Wizard PCR Purification Kit (Promega Corporation, WI, USA) and the DNA sequenced with BigDye Terminator Cycle Sequencing kit v3.1 (Applied Biosystems, Perkin-Elmer Corporation) using the manufacturer's instructions. Sequence matches were identified using BLASTn [16] at the NCBI BLASTn website [17] during April 2008.

### Design of microarray probes

The overall strategy was to design probes covering the major bacterial groups and dominant species present in the human GI tract, identified using both traditional culture based methods and molecular techniques [6,18-20]. Whenever possible, we designed at least two different probes for the same bacterium to confirm probe specificity. Probes were aimed to cover as wide a range of taxonomic hierarchy as possible. The selection of strains chosen for oligonucleotide design were based on their availability in a culture collection and access to their complete, or near-complete (1,000 bp or greater) 16S ribosomal gene sequence. Bacterial 16S ribosomal gene sequences were obtained via the NCBI Entrez-nucleotide website [21], the Ribosome Data Project 8.1 website [22,23] or from in-house sequencing.

Initially Array Designer 2.0 (PREMIER Biosoft International, Palo Alto, USA) was used to help create pilot microarrays to test optimum probe length, which consisted of 50-mer probes (see additional file 1), 40-mer probes (see additional file 2) and short nucleotide probes (mainly 16–21-mer) (see additional file 3). As well as specific bacterial oligonucleotides, random probes not expected to bind to any 16S ribosomal gene sequences were included in all three design platforms. Before experimental probe specificity testing, the probe sequences were checked for predicted specificity at the NCBI BLASTn website [17]. To perform this analysis, the low complexity filter was removed before submitting sequences to the BLASTn search. Short length oligonucleotides were also designed to determine the hybridisation and washing temperatures further, with zero, one and two base pair mismatches against a bacterial strain. These mismatch probes were designed by obtaining as many bifidobacterial 16S ribosomal gene sequences at the NCBI website [21] as possible, together with the *E. coli *16S ribosomal gene for referencing nucleotide position. These were aligned using the GeneDoc software [24], to identify regions of nucleotide variation and similarity. The alignment was subsequently used to find areas where single and duplicate mismatches occurred. A 15 nt dTTP spacer was added to the 5' end of the short length oligonucleotides to allow labelled DNA to bind more easily to the probe. The terminal 5' dTTP was amino-modified so the oligonucleotide could be chemically coupled to the microarray glass slide.

Specificity testing with genomic DNA from 17 pure bacterial cultures (Table 1) was initially used to evaluate performance of the three probe length variations (50-, 40-mer and short oligonucleotides). Specificity was defined as the total number of probes generating expected signals only when their corresponding 16S ribosomal gene was individually hybridised, divided by the total number of probes on the microarray. We confirmed the optimum hybridisation and washing temperatures through DNA melting curves. Following a successful trial of the 52 short nucleotide probes (see additional file 3), an expanded microarray was developed that contained 230 short probes (see additional file 4). This wide range of oligonucleotides was predicted with the Array Designer 2.0 software, identified via a literature search and from the probeBase website [25,26]. They are categorised into the following phylogenetic hierarchies: two universal, six phylum, three class, ten order, two family, seventeen genus and one hundred and sixty-two species. There were also sixteen probes covering clusters of bacteria and twelve controls.

### Printing of microarray probes

Oligonucleotide probes for microarray printing were purchased from Operon (Operon Biotechnologies, Cologne, Germany) and adjusted to 50 pmol μl^-1 ^in Quantifoil 1× spotting solution III (Quantifoil Micro Tools GmbH, Jena, Germany). Probes were printed onto epoxysilane coated glass slides (Schott Nexterion, Germany) in duplicate using a Stanford Style microarray spotter [27]. Each probe spot size was 100 μm when printed.

Epoxysilane coated glass slides were processed immediately prior to use. The slides were washed for 5 min in 0.1% Triton X-100 followed by two washes in 14 N HCl (Sigma-Aldrich, Poole, UK) pH 4.0, for 2 min. The slides were placed in 100 mM KCl for 10 min, washed twice in Milli-Q water for 1 min and immersed in blocking solution (50 mM ethanolamine (Sigma-Aldrich, Poole, UK) in 0.1 M Tris pH 9.0 at 50°C) for 15 min. Slides were transferred to a fresh solution of 0.1% Triton X-100 for 5 min and washed in 2× SSC, 0.2× SSC and Milli-Q water, each for 5 min. The slides were spun dry at 290 × *g *for 10 min, and then stored in the dark at room temperature until required. Slides were agitated at 250 rpm during all the steps and were not allowed to dry. All solutions were made with Milli-Q water.

### Fluorescence labelling of 16S ribosomal gene PCR fragments

Purified aliquots (2 ng) of 16S ribosomal gene PCR products obtained using Amp F and Amp R primers from pure bacterial cultures, or 200 ng of purified PCR product from the amplification of 16S ribosomal genes from faecal genomic DNA, were used as template for synthesis of Cy-dye labelled DNA products. The reaction contained 20 μl of 2.5 × random primer/reaction buffer mix from the Gibco Bioprime DNA labelling System (Invitrogen, UK). The solutions were heated for 5 min and then cooled on ice for 5 min. Subsequently 5 μl of 10 × dNTP mix (1.2 mM each of dATP, dGTP, dTTP; 0.6 mM dCTP; 10 mM Tris pH 8.0; 1 mM EDTA pH 8.0), 3 μl of 1 mM Cy5 dCTP or Cy3 dCTP (Amersham Biosciences, UK) and 1 μl of Klenow enzyme were added. The labelling reactions were incubated at 37°C for 5 h. The Cy3 and Cy5 reactions were combined where appropriate, and a Qia-quick PCR purification kit (Qiagen Ltd., UK) used to remove unincorporated Cy-dyes.

### Hybridisation and washing of epoxysilane coated glass slides

First, 10 μl of concentrated labelling solution was added to a mastermix of 1.5 μl 50× Denhardts solution, 2.25 μl of 20× SSC, 1.125 μl of *E. coli *tRNA (10 μg μl^-1^) and 0.375 μl of 1 M HEPES, pH 7.0. Then, 10% SDS (0.375 μl) was applied to the mixture and incubated at 100°C for 2 min. The mix was left to stand at room temperature for 5 min, centrifuged at 9,000 × *g *for 5 min and the supernatant transferred to a fresh tube. The microarray slide was placed in a hybridisation chamber (GeneMachines, CA, USA), labelling solution applied over the microarrays and a glass coverslip gently lowered on top of the solution to prevent it drying out. The slides were preheated on a hot block at 70°C for 30 min. Hybridisations were incubated for 15 h at 62°C, 63°C or 67°C for 40- and 50-mer probes and for short probes, temperatures between 55°C and 58°C were used.

The slides were placed in a slide transfer rack for movement between different washes and then directly submerged into 1 litre of wash solution containing 2× SSC and 0.1% SDS, and then gently shaken for 5 min. Several wash temperatures were tested, ranging from 65°C to 72°C for 40- and 50-mer probes and between 55°C and 66°C for shorter probes. Slides were gently agitated and racks transferred to a solution of 1× SSC and shaken at room temperature for 5 min at 250 rpm. This step was repeated once. Slides were then washed twice as described above in 0.2× SSC for 5 min and then spun dry at 290 × *g *for 5 min at room temperature. This optimised protocol is available at: .

### Scanning of microarray slides and data analysis

Slides were scanned using an Axon Genepix 4000A microarray scanner (Axon Instruments, Inc., California) with a scanning resolution of 5 μm. Image analysis was performed using Genepix Pro Version 5.0 (Axon Instruments, Inc., California). The data from Genepix were then exported to Excel for further processing. Microarray data analysis was carried out as follows: The local background signal was subtracted from the hybridisation signal of each separate spot; all values below ten were converted to ten to avoid negative values and the average of replicate spots was taken. The average signal obtained for the negative control spots was deducted from all replicate spot values. Any resulting negative number was made positive by converting to ten, which enabled further data analysis. Ten was chosen because this value would be far below the threshold of detection and would also not compromise further data analysis. Each spot was then divided by the total of all spots for the corresponding replicate. When comparing results from human faecal samples, the value from each spot was then divided by the average of all the thermophile reference spots obtained from the previous calculation. The resulting number represented the level of binding to a particular oligonucleotide which corresponded to a particular type or group of bacteria. At least two further microarray replicates were carried out to obtain the final average level of spot binding. Spots for which the average normalised binding intensity was equal to, or greater than 0.1, were considered to be positive. T-tests were performed to confirm significant differences between two samples.

### Minimum detection limits

*Salmonella enterica *serovar Typhimurium SL1344 cells were used to establish the sensitivity of the designed microarray. Cells were grown to an OD_600 _of 1.0 which corresponds to 8.8 × 10^8 ^cells ml^-1^. The culture was diluted in sterile PBS (8 gl^-1 ^NaCl, 0.2 gl^-1 ^KCl, 1.44 gl^-1 ^Na_2_PO_4 _and 0.24 gl^-1 ^KH_2_PO_4 _adjusted to pH 7.4) to provide a cell density in the range of 8.8 × 10^8 ^to 8.8 × 10^3 ^cells per ml. The dilutions were mixed with 1 g of fresh faecal sample and the DNA was subsequently extracted from 0.4 g of this mix. Furthermore, the serial dilutions were plated onto XLD plates (Oxoid, UK) containing 100 μg ml^-1 ^streptomycin in order to estimate the initial numbers of viable bacteria. The extracted genomic DNA from the *S*. Typhimurium spiked faecal samples was used as template for PCR. In the subsequent microarray experiments, the detection limit of the SAL455 probe was determined.

### Microarray data

The microarray dataset has been deposited in the ArrayExpress database , accession number: E-MEXP-1395.

## Results

### Probe design, phylogeny and specificity

A series of experiments with different hybridisation conditions were conducted, to examine the performance of microarrays with different probe lengths for detection of faecal bacteria. For microarrays with 50-mer probes (see additional file 1) and 40-mer probes (see additional file 2), we tested both hybridisation and washing temperatures ranging from 62°C to 72°C. A hybridisation temperature of 65°C combined with a first wash temperature of 72°C gave the best combination of signal intensity and specificity (see additional files 1 and 2). However, the level of specificity was variable. For most 50-mer probes tested with individually labelled 16S ribosomal genes from the seventeen reference strains (Table 1), the specificity ranged between order to species level, even though the probes were mainly designed to be specific at the genus or species level. A minority of the 50-mer probes were totally non-specific. The use of 40-mer oligonucleotide microarrays improved specificity whilst giving taxonomic discrimination at a range of hierarchies. We determined whether the addition of formamide to the hybridisation solution improved fidelity, as this has been reported to improve specificity [28]. Although the addition of formamide resulted in a general increase in signal intensity, the specificity was in fact reduced (unpublished data).

A short oligonucleotide (16-21-mer) pilot microarray with 52 probes was used to investigate the effectiveness of shorter probes. The validity of probes in the short pilot microarray were first assessed by hybridising them against individually labelled 16S ribosomal genes amplified from pure cultures (Table 1) of the corresponding bacterial species, at various hybridisation and washing temperatures. In general, specificity was achieved at either the species or genus level (see additional file 3). The binding efficiency of a target was highly dependent on the probe with some, such as *B. angulatum *and *B. pseud *&* catenulatum*, giving very low signals. After analysis of the specificity data, a cut-off value of 0.1 was defined as showing the presence of a specific bacterium, since below this value non-specific signals were obtained when DNA from pure cultures were tested. In general, the binding intensity (i.e. fluorescent signal) of all the short oligonucleotides was lower than the longer oligonucleotides. Nevertheless, the short oligonucleotides offered more accurate discrimination between various bacterial taxonomic hierarchies. When comparing the experimental microarray-based data versus the BLASTn predicted specificity, the short probes showed 84% specificity, whereas the longer probes were less specific: 66% for 50-mer probes (see additional file 1) and 75% for 40-mer probes (see additional file 2).

**Figure 1:**
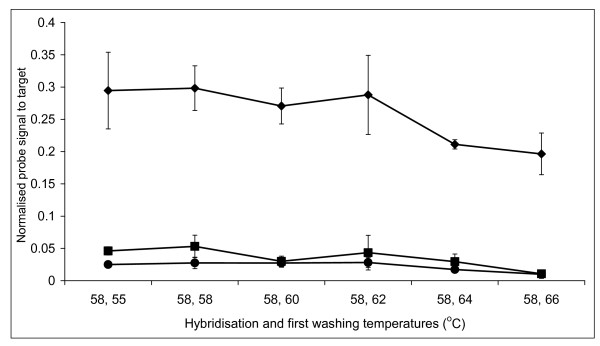
**Short oligonucleotide melting curve profile of the perfect, 1 nt and 2 nt mismatch probes**. Melting curve profile of the perfect match probe BLON135a (◆), 1 nt mismatch probe BLON135b (■) and 2 nt mismatch probe BLON135c (●) after hybridisation with fluorescently labelled 16S ribosomal DNA PCR products from *Bifidobacterium longum*. The error bars represent the standard deviation.

**Figure 2:**
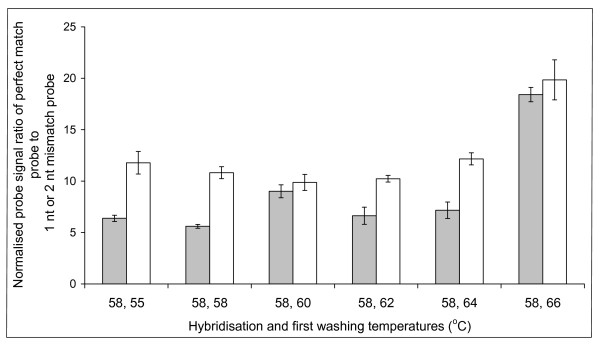
**Determination of the optimum washing temperature for the short oligonucleotide probes**. Discrimination of the perfect match probe (BLON135a), against either the 1 nt (BLON135b) (grey bars) or 2 nt (BLON135c) (white bars) mismatch probes. Labelled 16S ribosomal DNA from a pure culture of *Bifidobacterium longum *was hybridised at 58°C and washed between 55°C and 66°C. Perfect match probe intensity at each different temperature was then divided either by the 1 nt or 2 nt corresponding mismatch binding, and error bars represent the standard error.

In order to confirm the optimal hybridisation and washing conditions for the short probes, we performed DNA melting curves. The labelled 16S ribosomal gene from a pure culture of *Bifidobacterium longum *was hybridised to the corresponding probes containing 0, 1 or 2 nt mismatches. This established that hybridisation at 58°C gave the best discrimination (unpublished data). A range of washing temperatures were examined and again confirmed that 58°C was most selective for hybridisation (Figure 1 and Figure 2). As with the pure culture specificity testing, hybridisations and washes at higher temperatures resulted in greater specificity, but the intensity of the specific signal was reduced leading to increased noise.

To summarise, although short oligonucleotides generally gave lower signals than long oligonucleotides, their increased specificity facilitated a more accurate discrimination of bacterial groups at various taxonomic hierarchies. These findings were used to optimise the design of a community microarray which contained 230 short oligonucleotides (see additional file 4) that were used for analysis of human faecal samples.

### PCR amplification of faecal genomic DNA

For the purpose of diminishing the chance of PCR generated mismatches, high fidelity Phusion polymerase was used to amplify faecal genomic DNA by PCR. In order to minimise PCR amplification bias, only 15 PCR cycles were carried out. It has been reported that a 10 cycle PCR detects the greatest diversity of bacterial species [29], but 15 cycles were necessary to generate a 1.5 kb band that was visible in an agarose gel. This amplification procedure should help to maximise the accuracy of microarray data, since the DNA sequences and the proportions of 16S ribosomal genes obtained will have undergone minimal changes during PCR.

### Design of the internal standard

The short oligonucleotide community microarray incorporated an independent PCR amplification control step. For GI tract profiling, this was a novel approach which allowed all experiments to share a fixed reference point facilitating the comparison of data from any investigation. For all hybridisations, the spot binding intensity from *Thermus thermophilus *16S ribosomal genes was used as the control to normalise the data. *T. thermophilus *genomic DNA (10 pg) was amplified in the same 50 μl PCR reaction as each faecal genomic DNA template. The *T. thermophilus *DNA did not hybridise to any other spots on the microarray. This control spike allowed all data from replicate experiments to be normalised prior to comparison between samples from the same or different individuals. This novel approach enabled correlations of bacterial levels from separate experiments to be explored, providing useful information on gut bacterial diversity.

### Determination of the detection limit

**Figure 3:**
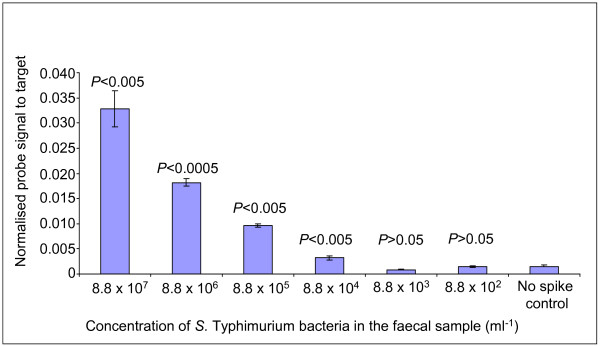
**Microarray detection limit profile using different concentrations of *Salmonella *cells spiked into a faecal sample**. Microarray detection limit profile for the *Salmonella *genus probe using serial dilutions of *S. enterica *serovar Typhimurium cells spiked into a human faecal sample. The standard error is represented by error bars. The *P *values are the degree of significance between the intensity recorded from a particular serial dilution and the intensity value obtained from the control sample with no spike.

*S. enterica *serovar Typhimurium SL1344 was used to establish the sensitivity of the short oligonucleotide community microarray. Absence of *S*. Typhimurium in a faecal sample was initially confirmed by plating. Subsequently the sample was spiked with various concentrations of *S*. Typhimurium cells prior to DNA extraction. The presence of *S*. Typhimurium at the correct levels in the spiked samples was confirmed by viable cell counts on XLD agar plates (unpublished data). The SAL455 probe detected *S*. Typhimurium in all samples which contained greater than 8.8 × 10^3^ cells ml^-1 ^(*P *< 0.05) (Figure 3). This detection limit is impressively low when compared to the total gut bacterial population of 1 × 10^12 ^to 1 × 10^13 ^cells g^-1^.

### Profiling of microarray signals from human samples

The short oligonucleotide microarray was used to determine microbial diversity within faecal samples from three healthy individuals, designated A, B and C (Table 3; Additional file 5). A hybridisation signal was recorded from ninety-four probes. Labelled DNA from all three individuals hybridised to the *B. longum *group and *B. bifidum *probes, indicating the presence of these organisms in the samples. The highest levels were recorded from individual A. Various representatives of the 'bacteroides' group were successfully identified by the microarray. Four different probes with specificity for *B. putredinis *DNA displayed the highest intensity in sample A. *B. thetaiotaomicron *and *B. ovatus *were also present in all three faecal samples. *B. vulgatus *was detected by the *B. vulgatus*1 & 2 probes. The levels of these bacteria among individuals varied for the two oligonucleotides, probably because the *B. vulgatus*1 probe had specificity to more uncultured microorganisms (according to BLASTn predictions). The presence of *B. merdae*, *B. stercoris *and *Flexibacter *spp. was identified in some samples (see additional file 5).

**Table 3:** Microarray binding levels of selected 16–21-mer microarray probes from healthy individuals and a UC patient

**Microarray Probe **	**Individual A**	**Individual B**	**Individual C**	**Disease**	**Remission**
*B. longum *grp1*	+++	+	+	++++++	+++

*B. longum *grp2*	++++	+	+	+++	++

*B. longum *grp3*	++++++	++	+	++++++	++++++

B. bifidium*	++++++	+	+	+	+

*B. thetaiotaomicron *&* B. ovatus*	++++++	++++	++++++	+	+++++

*B. ovatus*	+	+	+	-	+

*B. thetaiotaomicron*	++	+	+++	-	-

*B. vulgatus*1	++++++	+++	++++	+	++++++

*B. vulgatus*2	+	+	+	-	-

*B. putredinis*1	++++++	+	+	+	++

*B. putredinis*2	++++	+	+	-	+

*D. piger*2	-	-	-	-	+

*D. piger*4	-	-	-	+	++

Enterobacteriaceae1*	++++	++++++	++++++	++	+

Enterobacteriaceae2	-	+	+	-	-

CclusterXIVab*	++++++	++++	++++++	++	++

*C. coccoides*	++++++	++++++	++++++	++++	++

*C. clostridiformes*1	+++	+	++	+	++

*C. nexile*	+	+	+	-	-

*C. symbiosum*	+	+	+	-	-

CclusterIV	++	+	+	+	-

*C. leptum*2*	+	+	+	+	+

*C. butyricium*	+	+	+	-	-

*C. paraputrificum*	+	-	+	-	-

ClusterIII	++++++	++++++	+++	++	+

*E. cylindroides *clust1	++++++	++++	++++	+	+

*E. cylindroides *clust2	++++++	+++++	+++++	+++	+

*E. biforme*3	+	+	+	++	+

*E. biforme*4	+	+	+	+	+

*E. rectale*1	++	+	+	++	+

*E. rectale*2	+++++	+	++	+++	+

*R. lactaris*	++++	+	+	+	+

*R. torques*	+	-	-	-	-

*R. albus *&* R.flavefaciens*1	++	+	+	-	-

*R. albus *&* R. flavefaciens*2	++++++	++++	++	++	+

*R. callidus*	+	+	++	-	-

*R. bromii*	++++++	++	+++	-	-

Verrucomicrobiales	++	++++++	++++++	-	-

Microarray binding levels of selected 16–21-mer microarray probes from three healthy individuals and a UC sufferer during active disease and remission phases.

Hybridisation intensities, converted into symbols are: no signal to 0.09, -; 0.10 to 0.99, +; 1.00 to 1.99, ++; 2.00 to 2.99, +++; 3.00 to 3.99, ++++; 4.00 to 4.99, +++++; 5.00 or greater, ++++++. Probes highlighted with a * indicate they were used for initial specificity testing.

A number of clostridial species were detected in all faecal samples. Individual A had the highest levels of clostridia belonging to Clusters XIVab and IV. The members of Cluster XIVa that were detected included *Clostridium coccoides*, *C. clostridiformes*1, *C. nexile *and *C. symbiosum*. Bacteria identified belonging to Cluster IV, the second major dominant clostridial group, included *Clostridium leptum*, *C. butyricium *and *C. paraputrificum*. The Cluster III probe revealed that some bacteria belonging to this group were present in all samples.

Eubacteria were also identified, with most belonging to Cluster XIVa. *E. rectale *was represented by two *E. rectale *probes and both showed that individual A had the greatest number of this bacterium. *E. ventriosum *and *E. halii *were also detected in all three samples. Enterobacteriaceae were recorded from all three volunteers with individuals B and C having the greatest amount and A the least. Ruminococci probes detected members of this genus including *R. lactaris *and *R. torques *from Cluster XIVa and *R. albus*, *R. bromii*, *R. flavefaciens *plus *R. callidus *and from Cluster IV. Each Ruminococci probe produced different profiles for the three individuals.

Streptococci were identified, with individual A showing the highest level. There were three Streptococci probes specific for different species, however each individual's profile from these three probes were similar, indicating that the oligonucleotides may only give specificity to the genus level for this bacterial group. Members of the Verrucomicrobiales were present in high numbers in samples B and C. The *T. thermophilus *levels were consistent throughout and as expected, the negative controls produced no significant binding. The microarray data identified a diverse bacterial community inhabiting the GI tract of the three healthy individuals, and showed that the relative composition of the population is host specific.

### Bacterial diversity in a patient with Ulcerative Colitis (UC)

**Figure 4:**
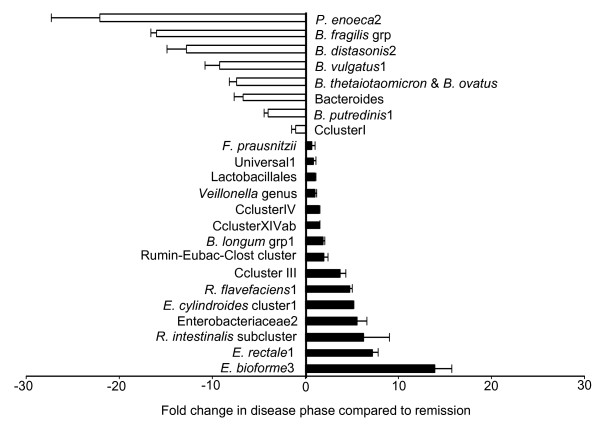
**GI tract bacterial changes in an Ulcerative Colitis patient during the disease state compared to remission**. Selection of bacterial probes showing the fold change in signal intensity during the disease phase compared to remission, in a patient suffering UC. Black bars indicate probes whose signal is greater in the disease state and white bars are those probes which are greater in remission. Probe identities for the black bars are to the left of zero on the x axis and white bar probe identities are to the right of zero. The error bars represent the standard error.

The short oligonucleotide community microarray was used to investigate the bacterial community of a patient suffering from UC (Figure 4; Table 3; Additional file 5). One sample was taken whilst the patient was suffering from a relapse, and the second six months later during remission. The results indicated a significant difference in the bacterial community from this single individual at two distinct times, and under different circumstances. One of the most noticeable changes in the population during the active disease phase, compared to remission, was the elevated levels of bacteria belonging to Clostridia clusters IV and XIV, particularly *E. biforme*, *E. rectale *and the *E. cylindroides *Clusters. Other Clostridia such as Clostridium Cluster I revealed no significant differences between samples taken during the active disease phase and remission. Of the ruminococci, only the *R. flavefaciens *and *R. albus *&* R. flavefaciens*2 probes demonstrated large increases during the active state. The *Roseburia intestinalis *sub-cluster exhibited a 6.2-fold elevation in intensity, while Rumin-Eubac-Clost Cluster levels increased by 1.9-fold, the difference probably reflecting the broader specificity of the latter probe. During the disease state, Enterobacteriaceae and the *Bifidobacterium longum *group were more abundant.

In addition to recording specific bacterial probes or groups of bacterial probes increased in the active disease phase compared to remission, other probes showed lower intensities in the active disease phase when compared to remission. In the case of 'Bacteroides', levels were reduced more than 10-fold for some species. The greatest reduction in a single probe representing a bacterium was *Prevotella enoeca*2 where a 22-fold lower level was observed. At both time points, there were some probes (*F. prausnitzii*, Lactobacillales, the *Veillonella *genus and the universal oligonucleotides), where no significant differences were observed between the two states.

### The varying intensities of microarray signals reflect gross levels of bacteria in faecal DNA from three healthy individuals

**Figure 5:**
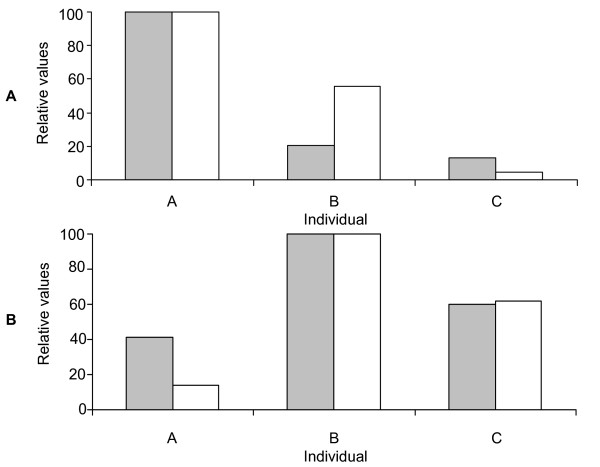
**Analysis of microarray and comparative PCR data**. **(A) **Analysis of the microarray (grey bars) and comparative PCR data (white bars) using *Bifidobacterium longum *grp3 probe and three healthy individuals' faecal flora (A-C). **(B) **Analysis of the microarray (grey bars) and comparative PCR data (white bars) using the Enterobacteriaceae1 probe and three healthy individuals' faecal flora (A-C). For both figures 5A and 5B, the data is displayed using relative values whereby the highest value for the two experiment types is used to compare intensities from the two other individuals.

A comparative PCR approach was used to determine whether the different normalised microarray signal intensities from the three healthy volunteers reflected their true abundance in the faecal genomic DNA. The PCR used one primer with the same sequence as the *B. longum *group3 microarray probe and a second primer also specific to *B. longum *(Table 2). Comparison of the microarray and PCR data (Figure 5A) confirmed that individual A had significantly higher levels of *B. longum*, and that individual C had the lowest levels of this bacterium (*P *< 0.05).

To further evaluate whether microarray data were valid, a second PCR of human faecal DNA was performed using a forward primer the same sequence as the Enterobacteriaceae1 probe (Figure 5B) and second primer specific to Enterobacteriaceae (Table 2). Relative values obtained showed identical trends in the two techniques. In contrast to the *B. longum *results, individual B had the highest signal and A the lowest. All PCR amplification profiles were significantly different from each other and the microarray data exhibited a significant difference between individuals A and B (*P *< 0.05). The microarray method was unable to significantly distinguish between C and A (*P *> 0.05), although the same trend, as observed in the PCR results, was apparent. This suggests that the microarray analysis discriminates effectively between large variations in bacterial numbers among samples. With further replicates, smaller differences between the levels of bacterial 16S ribosomal genes that bind to the same probe may also be distinguished.

### Comparison of microarray data with results from Denaturing Gradient Gel Electrophoresis (DGGE)

DGGE is a genetic fingerprinting technique that separates DNA on the basis of sequence variation into distinct bands on a gel. The identity of the bacterial species associated with individual bands can be determined by the purification and sequencing of selected bands. This molecular technique was used to profile the faecal microbiota from the three healthy individuals and the UC patient during both disease and remission phases. Using this approach provided a comparison of data from the short oligonucleotide microarray with those from an established molecular profiling method.

#### Comparison of data from healthy individuals

**Figure 6:**
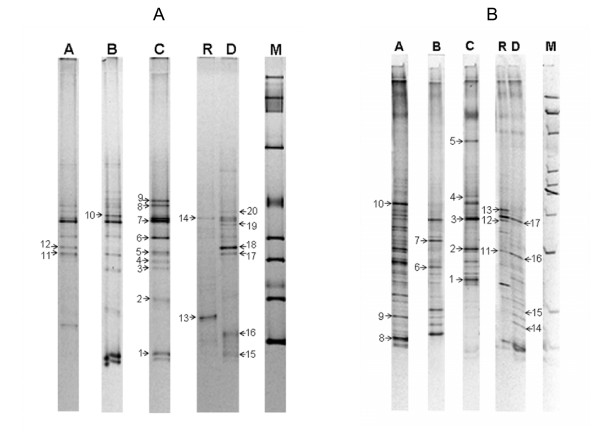
**Faecal bacterial community analysis using DGGE**. **(A) **DGGE analysis, in a denaturing gradient of 40–60% urea and formamide, of the PCR products from the amplification of faecal bacterial genomic DNA from three healthy individuals (A, B and C) and a UC patient in active (D) and remission (R) states of the disease, using primers for 16S ribosomal gene region V6-V8. **(B) **DGGE analysis, in a denaturing gradient of 30–60% urea and formamide, of the PCR products from the amplification of faecal bacterial genomic DNA from three healthy individuals (A, B and C) and a UC patient in active (D) and remission (R) states of the disease, using primers for 16S rDNA region V3. For both gels, numbered arrows point to bands subjected to re-amplification and sequencing, and correspond to the amplicon identities in Table 4 (V6-V8) and Table 5 (V3). The ladder on each gel is used for quality control purposes only.

Primers targeting the V6-V8 region of the 16S ribosomal gene, were used to amplify a DNA fragment from the total microbial community DNA in the three healthy individuals' samples used for microarray analysis. A large proportion of the DGGE bands in figures 6A and 6B were picked and the identity of those which were successfully PCR amplified and sequenced are shown in Tables 4 and 5. Six bands were clearly identified as being derived from specific known organisms (Figure 6A; Table 4). Both the microarray data and the DGGE showed that individuals B and C carried *Akkermansia muciniphila*, a member of the Verrucomicrobiales (Table 4, band 1). A band corresponding to this organism was absent in individual A, consistent with the very low levels revealed using the microarray. *Eubacterium rectale *was present at the highest level in individual A according to the microarray data. This was confirmed by DGGE, with a band in sample A identified as *E. rectale *(Table 4, band 12). Signals from the microarray indicated the low abundance of this organism in samples B and C and a DGGE band representing this organism was absent in samples from these individuals. The DGGE band that closely matched *R. faecalis *(Table 4, band 4), could not be directly related to the microarray data as there was no unique probe for this organism.

**Table 4:** Identities of variable region V6-V8 DGGE bands picked and sequenced from the gel in Figure 6A

**DGGE band number**	**Closest relative (if known)**	**Origin and accession number**	**Sequence homology (%)**
1	*Akkermansia muciniphila*	Adult human gut [GenBank:AY271254]	97

2	Uncultured human gut bacterial clone	Elderly human [GenBank:AY920038]	100

3	Uncultured human gut bacterium	Human gut [GenBank:EF402349]	99

4	*Roseburia faecalis*	Healthy adult faecal sample [GenBank:DQ144129]	99

5	Uncultured human gut bacterial clone	Human adult faecal sample [GenBank:DQ795140]	98

6	Uncultured bacterial clone	Rumen [GenBank:DQ394592]	96

7	Uncultured Clostridiales clone	Human gut [GenBank:EU530233]	99

8	*Lachnobacterium bovis*	Animal rumen [GenBank:AF298665]	100

9	*Lachnospira pectinoschiza*	Greenland ice core [GenBank:AY169414]	98

10	*Butyrivibrio crossotus*	No origin given [GenBank:X89981]	98

11	Uncultured bacterial clone	Human adult faecal sample [GenBank:AY916377]	99

12	*Eubacterium rectale*	Healthy human gut [GenBank:AY804151]	99

13	Uncultured bacterial clone	Porcine intestine [GenBank:AF371769]	100

14	Uncultured bacterial clone	Human Crohn's disease patient [GenBank:AF530350]	99

15	*Bifidobacterium longum *NCC2705	Human intestinal tract [GenBank:AE014295]	100

16	Uncultured bacterial clone	Animal rumen [GenBank:DQ394592]	97

17	Uncultured bacterial clone	Human gut [GenBank:AF132248]	94

18	*Eubacterium rectale*	Healthy human gut [GenBank:AY804151]	99

19	Uncultured bacterial clone	Crohn's disease patient [GenBank:AF530350]	97

20	Uncultured bacterial clone	Elderly human [GenBank:AY920002]	99

**Table 5:** Identities of variable region V3 DGGE bands picked and sequenced from the gel in Figure 6B

**DGGE band number**	**Closest relative (if known)**	**Origin and accession number**	**Sequence homology (%)**
1	*Barnesiella intestinihominis*	Human faeces [GenBank:AB370251]	100

2	Uncultured bacterial clone	Adult human faeces [GenBank:AY983922]	100

3	*Bacteroides dorei*	Human faeces [GenBank:AB242142]	100

4	Uncultured Bacteroides clone	Human faeces [GenBank:EU381174]	100

5	*Bacteroides fragilis*	No origin given [GenBank:CR626927]	100

6	*Desulfovibrio *spp.	No origin given [GenBank:U60095]	100

7	Uncultured bacterium clone	Human faeces [GenBank:DQ800759]	100

8	Uncultured faecal bacterium clone	Adult with Crohn's disease [GenBank:AY471705]	100

9	Uncultured bacterium clone	Human adult faecal sample [GenBank:AY985184]	100

10	*Bacteroides vulgatus*	Human large intestine [GenBank:AY978412]	99

11	Uncultured Bacteroides spp.	Human faeces [GenBank:DQ808592]	100

12	*Bacteroides eggerthii*	Human intestine [GenBank:AB050107]	100

13	Uncultured bacterial clone	Activated sludge in circulation flush toilet [GenBank:AB196029]	95

14	*Prevotella *spp.	Human oral clone [GenBank:AY005054]	92

15	Uncultured bacterial clone	Human faeces [GenBank:AY343236]	97

16	*Bacteroides uniformis*	Human faeces [GenBank:AB247142]	100

17	*Bacteroides eggerthii*	Human faeces [GenBank:AB050107]	100

Further experiments comparing DGGE results with the microarray data were carried out using primers targeting the V3 region of the 16S ribosomal gene. From a total of ten bands sequenced, four corresponded to Bacteroides, while many had highest sequence homologies to unknown bacteria (Figure 6B; Table 5). The Bacteroides species identified did not have corresponding species-specific probes on the microarray. A *B. fragilis *group oligonucleotide, which included specificity to *B. fragilis*, *B. thetaiotaomicron*, *B. vulgatus*, *B. eggerthii *and *B. uniformis *was present, and both the microarray and the DGGE results suggest that individual B has lower levels of bacteria from this group. The DGGE recorded the presence of a member of the *Desulfovibrio *genus in individual B (Table 5, band 6), but this result cannot be directly compared to microarray data since the microarray only had probes targeted for *D. piger*.

#### Comparison of data using the two samples from the UC patient

A similar DGGE analysis and comparison with the microarray data was undertaken for the two samples obtained from the UC patient during the disease and remission states (Figure 6A; Figure 6B; Table 4; Table 5). Sequence analysis of band 18 from the DGGE separation of the V6-V8 region was identified as *E. rectale *(Figure 6A; Table 4). This band was only detected during the active disease state. Similarly, the microarray data indicated that *E. rectale *was present in considerably higher levels in the active disease phase (up to 7.2-fold increase) when compared with remission. The fact that DGGE did not reveal an equivalent *E. rectale *band in the remission sample probably reflects the relatively low sensitivity of this molecular profiling method. Another DGGE band was identified as *Bifidobacterium longum *and had elevated levels in the diseased state. Again this is consistent with data derived from the microarray analysis. Interestingly, two of the DGGE bands corresponding to unidentified bacteria most closely matched 16S ribosomal gene sequences previously detected in patients with Crohn's disease (CD) (Figure 6A; Table 4, bands 14 and 19).

DGGE separation of the V3 region of the 16S ribosomal gene and the sequencing of selected bands revealed the presence of 'Bacteroides' species (Figure 6B; Table 5). *B. fragilis *and *B. vulgatus *bands that were detected in the healthy individuals were absent in the case of the UC patient. Very similar results were obtained using the microarray, especially for the active disease state sample. However, only the microarray analysis revealed that levels of 'Bacteroides' species appeared to recover during remission. This difference probably reflects the greater sensitivity of the microarray-based approach. In summary, we have shown there is a close correlation of results between the microarray and DGGE technique when studying the faecal bacterial population from three healthy individuals and a patient with UC.

## Discussion

In this study we have developed a short oligonucleotide community DNA microarray for the detection of members of the human gut microbiota. Using this microarray we identified differences within the GI tract bacterial community from healthy and diseased individuals. Data sets were validated by analysis of the same samples using DGGE and PCR.

Initially, 50-mer probes were tested and generally a 10% sequence divergence was required to distinguish between specific and non-specific hybridisation. Typically, when stringent hybridisation and washing temperatures were employed, a 4 nt difference was sufficient to differentiate between genera but not species. For example, the *Bifidobacterium pseudocatenulatum *probe (BPSE1438) with 4 nt difference to most other bifidobacterial 16S ribosomal DNA genes, could not differentiate at the species level. This was also true for the *Bacteroides vulgatus *probe (BVUL1146) to *Bacteroides fragilis *16S ribosomal DNA. For 40-mer probes, a higher specificity was obtained with as little as a single base difference required for species discrimination (for example the *B. longum *probe BLON451 to other bifidobacterial 16S ribosomal DNA genes), although most probes required at least a 3 nt difference. Previous reports have suggested that probes with large numbers of mismatches or internal single nucleotide mismatches, exhibited higher specificities compared to mismatches in the first or last nucleotide of the probe [30]. Furthermore, avoiding runs of complementary nucleotides of 10–16 nt also increases specificity [31]. These points were used in the design of both short and long oligonucleotide probes in our study.

Shorter oligonucleotides (16–21-mer) were also evaluated. Initially these probes were designed to be a uniform length, as used in an earlier study [32], but this approach was unsuccessful due to the large number of GI tract microorganisms with similar 16S ribosomal gene sequences. Instead, a compromise was made whereby a 47°C–52°C nearest neighbour melting temperature was selected. In this case the minimum difference required to distinguish bacteria was 1 nt. For example the *B. angulatum *16S ribosomal gene product did not cross-hybridise to the *B. adolescentis *probe, and this differentiation was comparable to the result of an earlier study [33]. However, a 2 nt difference was preferred as some probes selected for differentiation between species with a single 1 nt difference only showed family level specificity.

Hybridisation of the 16S ribosomal gene PCR product from 17 bacterial reference strains revealed that 84% of oligonucleotides showed the predicted binding pattern. As with other studies, the shorter length probes showed higher specificity [34], but a much reduced signal strength compared to longer oligonucleotides [30]. Lower specificity of the longer probes results from the higher melting temperature probes (i.e. generally those with longer nucleotide lengths) which are capable of capturing non-specific targets, mainly due to their stronger affinities arising from the greater number of guanine and cytosine bases in their sequences [35]. Shorter probes show a higher specificity because less opportunity exists for non-specific binding as a result of their reduced length and lower melting temperature. Our short probes offered the optimum choice for profiling the human GI tract community because less point mutations were required to achieve the same or greater differentiation between bacterial groups/species compared to 50- and 40-mer probes. The final selection of our probes reflected a trade-off between signal strength and fidelity. A cut-off value of 0.1 was defined using pure culture bacterial DNA, below which non-specific signals were generated. A similar approach was developed for a study designed to detect sulphate reducing prokaryotes [32]. *T. thermophilus *DNA was used as an independent control to normalise data. Adding *T. thermophilus *genomic DNA did not interfere with the PCR amplification of bacterial 16S ribosomal genes from the faecal genomic DNA. This was confirmed by DGGE analysis of the same faecal sample with or without a thermophile spike using DGGE primers targeting variable region V6-V8 (unpublished data). This independent control offers a novel way to normalise gut profiling data and allowed multiple experiments from different samples to be compared.

Using the optimised microarray protocol, the detection limit for the microarray is 8.8 × 10^4 ^bacteria per gram of faecal sample (Figure 3) from a total population of around 1 × 10^13 ^bacteria per gram of faecal contents. This is a very low detection limit showing greater sensitivity than other techniques applied to the profiling of microbial communities. For example, DGGE can only detect bacteria constituting more than 1% of the complete community and in the case of FISH the detection level is around 1.0 × 10^6^ cells [36]. Our detection limit is similar to that reported for another oligonucleotide microarray that has been designed to study the microbial community of compost [37]. A good example of the differences in sensitivity in our study was the comparison of *E. rectale *levels in the UC patient using DGGE and the community microarray. A similar combination of DGGE and microarray profiling has been used by other authors with equal effectiveness [38,39]. The DGGE data confirms that the microarray probes for Verrucomicrobiales, *E. rectale *and *B. longum *were specific. Analysis of the microarray and comparative PCR results indicated that the microarray binding intensity showed very similar trends to the amplification profile of the corresponding PCR, when using the same sequence microarray probe as one of the PCR primers.

The optimised microarray procedure revealed differences in the types and levels of bacteria present in faecal samples from three healthy individuals. Data were generated for Bacteroides, Eubacteria, Bifidobacteria, Clostridia, Enterobacteriaceae, Lactobacilli, Ruminococci, Streptococci and Verrucomicrobiales, all of which have previously been identified in the human GI tract [6,40-42]. These findings, together with validation and cross-referencing using other molecular approaches, confirmed that our procedure can be accurately used to study the GI tract microbiota. Further development, including the incorporation of additional specific probes, will enhance the use of this microarray. One area where the coverage could be improved is in the detection of bacteria such as *Porphyromonas *and *Prevotella*, which were barely recorded in this study compared to previous findings [43]. This low level of detection may reflect biases in the extraction of specific bacterial genomic DNA or limitations associated with the binding specificity of probes and PCR primers. Data from the literature indicates that it is not always possible to predict the hybridisation characteristics of oligonucleotide probes for species discrimination [44]. In this study we maximised the chances of specificity by avoiding complementary runs of nucleotides, similar to another reported approach [31].

It is estimated that more than one million Americans have UC or Crohn's disease (CD), the two most common forms of Inflammatory Bowel Disease (IBD) [45]. The inflammation in the bowel caused by IBD can be debilitating. Literature suggests the disease may have genetic and immunological factors, environmental influences and the possible involvement of indigenous gut microbes [46-49]. In our study, two faecal samples from a UC patient were taken six months apart. The community microarray showed significant differences in the levels of Bacteroides, Eubacteria and sulphate reducing bacteria. The sequencing of DNA bands separated by DGGE broadly confirmed the microarray results. In this patient, bacterial population levels, especially from species such as Eubacteria, were different in the disease and remission states. This suggests that bacterial community instability may be a cause or it may be a consequence of other factors in the disease. Two of the biggest differences occurred in the levels of *E. rectale *and *E. biforme*. A large reduction in the levels of Bacteroides (and the sole *Prevotella *probe to give a signal) in the disease phase was observed when compared both to the remission phase of the same individual and to the three healthy individuals. This trend has been identified by others [50]. Sulphate reducing bacteria (SRB) have been suggested as a possible causative agent of UC. However it is not clear whether it is the presence or levels of these bacteria that is the cause of the disease, or a consequence of it. In one study the counts and carriage rates of SRB between healthy and diseased individuals showed no difference [51], while a second found them significantly higher in IBD patients compared to healthy individuals [49]. Our investigation also found that *Desulfovibrio piger *was significantly higher in both the disease and remission samples, compared to the samples from the healthy volunteers.

## Conclusion

We have developed an oligonucleotide based microarray for the rapid analysis of the gut bacterial community, giving an alternative means of profiling the human GI tract bacterial population. We tested different length probes and discovered that shorter oligonucleotides (16–21-mer) showed the greatest specificity. We designed an expanded short oligonucleotide microarray, comprising of 230 probes targeting species or groups of GI tract bacteria. This microarray was highly sensitive and detected less than 8.8 × 10^4 ^cells per gram of faecal contents. The utility of the designed microarray was tested by profiling gut microbial communities in healthy volunteers as well as in a UC patient during active and remission states. The microarray data were confirmed by cross referencing with other established molecular methods, and showed a diverse and dynamic community. Further probes will be added to the microarray, to include new sequences that have recently become available.

## Authors' contributions

CRH designed the study, participated in most of the techniques described in the paper and drafted the manuscript. SL helped with microarray design and data analysis. He also provided project ideas and contributed to the manuscript. KPR conducted DGGE experiments, supplied project ideas and helped draft the manuscript. UW carried out sequencing reactions, assisted in design of probes, provided project ideas and helped draft the manuscript. TJE participated in initial project design, gave study ideas and supervised part of the laboratory work. JCDH helped design the microarray, contributed project ideas and assisted with drafting of the manuscript. AN supplied project ideas, supervised part of the laboratory work and assisted with drafting the manuscript. MJG initiated the study, provided project ideas and assisted with drafting the manuscript. All authors read and approved the manuscript.

## Supplementary Material

Additional file 1 **Target organisms for 50-mer probes and actual tested microarray specificity using optimised hybridisation and washing conditions**. Complete list of 50-mer probes used in this study with tested specificity results described for the optimum hybridisation and first washing temperatures of 65°C and 72°C respectively. Click here for file

Additional file 2 **Target organisms for 40-mer probes and actual tested microarray specificity using optimum hybridisation and washing conditions**. Complete list of 40-mer probes used in this study with tested specificity results described for the optimum hybridisation and first washing temperatures of 65°C and 72°C respectively. Click here for file

Additional file 3 **Target organisms for 16–21-mer probes and actual tested microarray specificity**. Fifty-two short oligonucleotides (16–21-mer) selected for the pilot microarray. Described specificity testing results are for the optimum hybridisation and first washing temperatures of 58°C and 58°C respectively. Click here for file

Additional file 4 **Oligonucleotide probes, mainly between 16- and 21-mer in length, used for the expanded community microarray**. Unique oligonucleotide probes mainly 16–21-mer in length designed for the short community microarray. Complete list of 230 short oligonucleotide probes used in this study including the predicted specificity. Click here for file

Additional file 5 **Microarray binding levels of the entire 16–21-mer microarray probes from healthy individuals and a UC sufferer**. Complete set of normalised data values from three healthy individuals (A, B and C) and an Ulcerative Colitis patient in the active disease and remission states. Entire list of binding levels from the short oligonucleotide probes for healthy individuals (designated A, B, C) and the UC sufferer in the disease and remission states. Click here for file
