# Supplementary material for: A short-oligonucleotide microarray that allows improved detection of gastrointestinal tract microbial communities
Source: BMC Microbiol. 2008 Nov 11;8:195. doi: 10.1186/1471-2180-8-195 (PMC2628385; doi:10.1186/1471-2180-8-195)
Supplement: Additional file 1 — Target organisms for 50-mer probes and actual tested microarray specificity using optimised hybridisation and washing conditions. Complete list of 50-mer probes used in this study with tested specificity results described for the optimum hybridisation and first washing temperatures of 65°C and 72°C respectively. [file 1471-2180-8-195-S1.pdf]

Additional file 1 - Target organisms for 50-mer probes and actual tested microarray specificity using optimised hybridisation and washing conditions

Probe name[a]	Full name[a]	Target organism(s)[b]	Sequence (5'-3')	Hybridisation with pure culture[c]	Actual specificity
BANG1240	S-G-Bact-1240-a-S-50	*Bifidobacterium angulatum*, *B. minimum*, *B. scardovii*, *B. pullorum*, *B. merycicum*, *B. gallinarum*, *B. indicum*, *B. catenulatum*, *B. coryneforme*, *B. saeculare* and *B. subtile*	GCTACAATGGCCGGTACAACGGGAT GCGACATGGCGACATGGAGCGGATC	FP	Non-specific
BPSE1438	S-S-Bact-1438-a-S-50	*Bifidobacterium pseudocatenulatum*	CCGAAGCCGGTGGCCTAACCCTTTGT GGATGGAGCCGTCTAAGGTGAGAC	FP	Genus
BINF166	S-S-Bact-0166-a-S-50	*Bifidobacterium infantis*, *B. longum* and *B. suis*	GGTGGTAATGCCGGATGTTCCAGTTG ATCGCATGGTCTTCTGGGAAACGT	+	Species
BLON85	S-G-Bact-0085-a-S-50	*Bifidobacterium longum*, *B. infantis*, *B. suis*, *B. bifidum* and *B. breve*	CTTGCTTGGTGGTGAGAGTGGCGAAC GGGTGAGTAATGCGTGACCGACCT	FP	Genus
BADO198	S-S-Bact-0198-a-S-50	*Bifidobacterium adolescentis* and *B. ruminantium*	GCATGTCCTTCTGGGAAAGATTCTAT CGGTATGGGATGGGGTCGCGTCCT	FP	Non-specific
BFRA702	S-G-Bact-0702-a-S-50	*Bacteroides fragilis*, *B. thetaiotaomicron* and *B. acidifaciens*	TGCTTAGATATCACGAAGAACTCCGA TTGCGAAGGCAGCTCACTGGACTG	+	Species

Probe name	Full name[a]	Target organism(s)[b]	Sequence (5'-3')	Hybridisation with pure culture[c]	Actual specificity
BFRA583	S-S-Bact-0583-a-S-50	*Bacteroides fragilis*	AGCGTAGGTGGACTGGTAAGTCAGT TGTGAAAGTTTGCGGCTCAACCGTA	+	Species
BVUL1146	S-S-Bact-1146-a-S-50	*Bacteroides vulgatus*	TATGCTGAGGACTCTGACAAGACTGC CATCGTAAGATGTGAGGAAGGTGG	FP	Genus
BROI398	S-G-Bact-0398-a-S-50	*Bacteroides acidifaciens*, *B. thetaiotaomicron*, *B. eggerthii*, *B. fragilis*, *B. caccae*, *B. vulgatus*, *B. uniformis*, *B. stercoris* and *Prevotella heparinolytica*	ACCAGCCAAGTAGCGTGAAGGATGA CTGCCCTATGGGTTGTAAACTTCTT	-	No signal generated
CLEP575	S-S-Bact-0575-a-S-50	*Clostridium leptum*	GGGTGTAAAGGGTGCGTAGGCGGCG AGGCAAGTCAGGCGTGAAATCTATG	+	Species
CLEP725	S-S-Bact-0725-a-S-50	*Clostridium leptum*	CTTTAACTGACGCTGAAGCACGAAA GCATGGGTAGCAAACAGGATTAGAT	+	Species
EFAEC712	S-F-Bact-0712-a-S-50	Enterococcaceae	GTGAAATGCGTAGATATATGGAGGA ACACCAGTGGCGAAGGCGGCTCTCT	FP	Order
EFAE647	S-C-Bact-0647-a-S-50	Mostly Enterococcaceae	GGGAGGGTCATTGGAAACTGGGAGA CTTGAGTGCAGAAGAGGAGAGTGGA	+	Family
LACT704	S-G-Bact-0704-a-S-50	Lactobacilli	GTGGAATGCGTAGATATATGGAAGA ACACCAGTGGCGAAGGCGGCTCTCT	FP	Order
LACT812	S-G-Bact-0812-a-S-50	Lactobacilli	GGTAGTCCATGCCGTAAACGATGAG TGCTAAGTGTTGGGAGGTTTCCGCC	FP	Order

Probe name	Full name[a]	Target organism(s)[b]	Sequence (5'-3')	Hybridisation with pure culture[c]	Actual specificity
LJOHN71	S-S-Bact-0071-a-S-50	*Lactobacillus johnsonii*	GCACTAAATGAAACTAGATACAAGC GAGCGGCGGACGGGTGAGTAACACG	FP	Non-specific
STRE797	S-P-Bact-0797-a-S-50	Most streptococci and some other members of the Firmicutes	AGGATTAGATACCCTGGTAGTCCACG CCGTAAACGATGAGTGCTAGGTGT	+	Order
STRE1475	S-O-Bact-1475-a-S-50	Most streptococci and some *Enterococcus* spp.	GCCGCCTAAGGTGGGATAGATGATT GGGGTGAAGTCGTAACAAGGTAGCC	+	Order
ENTE1004	S-F-Bact-1004-a-S-50	Enterobacteriaceae and *Shewanella putrefaciens*	AACTTTCCAGAGATGGATTGGTGCCT TCGGGAACTGTGAGACAGGTGCTG	+	Family
ECOL83	S-F-Bact-0083-a-S-50	*Escherichia coli* and *Salmonella* spp.	CTTGCTCTTTGCTGACGAGTGGCGGA CGGGTGAGTAATGTCTGGGAAACT	+	Family
ECOLI83	S-F-Bact-0083-a-S-50	*Escherichia coli* and *Shigella boydii*	CTTGCTTCTTTGCTGACGAGTGGCGG ACGGGTGAGTAATGTCTGGGAAAC	+	Family
SALM1123	S-G-Bact-1123-a-S-50	*Salmonella* spp.	TGTTGCCAGCGATTAGGTCGGGAACT CAAAGGAGACTGCCAGTGATAAAC	N/T	No *E. coli* 16S ribosomal DNA binding
SALM809	S-F-Bact-0809-a-S-50	*Salmonella* spp. and *Enterobacter agglomerans*	GCCGTAAACGATGTCTACTTGGAGGT TGTGCCCTTGAGGCGTGGCTTCCG	N/T	No *E. coli* 16S ribosomal DNA binding

Probe name	Full name[a]	Target organism(s)[b]	Sequence (5'-3')	Hybridisation with pure culture[c]	Actual specificity
SHIG83	S-F-Bact-0083-a-S-50	*Shigella* spp. and *Escherichia coli*	CTTGCTGTTTCGCTGACGAGTGGCGG ACGGGTGAGTAATGTCTGGGAAAC	+	Family
ROB810	S-S-Bact-0810-a-S-50	*Ruminococcus obeum*	CTGGTAGTCCACGCCGTAAACGATG ATTACTAGGTGTTGGGGAGCAAAGC	+	Species
POSd1055	S-*-Bact-1055-a-S-50	Most bacteria	ATGGYYGTCGTCAGCTCGTGCCGTGA GGTGTYGGCTTAAGTGCCATAACGA	+	Domain bacteria
POSe1055	S-*-Bact-1055-a-S-50	Most bacteria	ATGGYYGTCGTCAGCTCGTGTCGTGA GATGTYGGGTTAAGTCCYGCAACGA	+	Domain bacteria
POSf1055	S-*-Bact-1055-a-S-50	Most bacteria	ATGGYYGTCGTCAGCTCGTGTTGTGA AATGTYGGGTTAAGTCCCGCAACGA	+	Domain bacteria
TMAR162	S-G-Bact-0162-a-S-50	*Thermotoga maritima* and *T. neapolitana*	GGGAAACCCTGGTTAATACCCCATAC GCTCCATCAACGCAAGTTGGTGGA	-	No signal from any labelled DNA recorded
SCER0646	S-G-Euk-S.cere-0646(*S. cerevisiae* strain CICC1308)-a-S-50	*Saccharomyces cerevisiae* and *S. bayanus*	CCTTTCCTTCTGGCTAACCTTGAGTC CTTGTGGCTCTTGGCGAACCGGGA	-	No signal from any labelled DNA recorded

[a] Nomenclature of the oligonucleotide probe according to Alm et al. [57].

[b] Target organism(s) of the probe from the BLASTn prediction [17].

[c] Hybridisation with pure culture results: +, positive hybridisation; -, no hybridisation; NT, not tested; FP, false positive.
