# Supplementary material for: A short-oligonucleotide microarray that allows improved detection of gastrointestinal tract microbial communities
Source: BMC Microbiol. 2008 Nov 11;8:195. doi: 10.1186/1471-2180-8-195 (PMC2628385; doi:10.1186/1471-2180-8-195)
Supplement: Additional file 2 — Target organisms for 40-mer probes and actual tested microarray specificity using optimum hybridisation and washing conditions. Complete list of 40-mer probes used in this study with tested specificity results described for the optimum hybridisation and first washing temperatures of 65°C and 72°C respectively. [file 1471-2180-8-195-S2.pdf]

**Additional file 2** - Target organisms for 40-mer probes and actual tested microarray specificity using optimum hybridisation and washing conditions

| Probe name | Full name[a] | Target organism(s)[b] | Sequence (5'-3') | Hybridisation with pure culture[c] | Actual specificity |
|---|---|---|---|---|---|
| BINF177 | S-St-Bact-0177-a-S-40 | *Bifidobacterium infantis* | CGGATGTTCCAGTTGATCGCAT GGTCTTCTGGGAAACGTT | + | Species |
| BLON451 | S-S-Bact-0451-a-S-40 | *Bifidobacterium longum* and *B. infantis* | GGAGCAAGCGAGAGTGAGTTT ACCCGTTGAATAAGCACCG | + | Species |
| BPSE996 | S-S-Bact-0996-a-S-40 | *Bifidobacterium pseudocatenulatum* | TTGACATGTTCCCGACAGCCGT AGAGATATGGCCTCCTTC | NT | No signal |
| BFRA400 | S-G-Bact-0400-a-S-40 | *Bacteroides fragilis* and *B. ovatus* | CAAGTAGCGTGAAGGATGAAG GCTCTATGGGTCGTAAACT | + | Species |
| BFRA561 | S-S-Bact-0561-a-S-40 | *Bacteroides fragilis* | CGGATTTATTGGGTTTAAAGGG AGCGTAGGTGGACTGGTA | + | Species |
| BVUL1144 | S-St-Bact-1144-a-S-40 | *Bacteroides vulgatus* | GGTTATGCTGAGGACTCTGACA AGACTGCCATCGTAAGAT | FP | Genus |
| BVUL588 | S-S-Bact-0588-a-S-40 | *Bacteroides vulgatus* | GCGTAGATGGATGTTTAAGTCA GTTGTGAAAGTTTGCGGC | FP | Genus |
| CLEP647 | S-S-Bact-0647-a-S-40 | *Clostridium leptum* | TGAAACTGTCTTGCTTGAGTGA AGTAGAGGTAGGCGGAAT | + | Species |
| CLEP765 | S-S-Bact-0765-a-S-40 | *Clostridium leptum* | CTGAAGCACGAAAGCATGGGT AGCAAACAGGATTAGATAC | + | Species |

| Probe name | Full name[a] | Target organism(s)[b] | Sequence (5'-3') | Hybridisation with pure culture[c] | Actual specificity |
|---|---|---|---|---|---|
| EFAE486 | S-G-Bact-0486-a-S-40 | *Enterococcus faecium*, *E. durans*, *E. hirae*, *E. faecalis*, *E. villorum*, *E. ratti* and *E. azikeevi* | AACTGTTCATCCCTTGACGGTA TCTAACCAGAAAGCCACG | FP | Order |
| FIRM712 | S-P-Bact-0712-a-S-40 | Firmicutes phyla | GTGAAATGCGTAGATATATGG AGGAACACCAGTGGCGAAG | + | Phylum |
| LCRI140 | S-G-Bact-0140-a-S-40 | *Lactobacillus crispatus*, *L. acidophilus*, *L. helveticus* and *L. jensenii* | CATAGTCTGGGATACCACTTGG AAACAGGTGCTAATACCG | NT | No signal generated |
| LCRI432 | S-G-Bact-0432-a-S-40 | *Lactobacillus crispatus*, *L. acidophilus*, *L. helveticus L. delbrueckii*, *L. suntoryeus*, *L. intestinalis*, *L. hamsteri*, *L. kitasatonis*, *L. psittaci*, *L. lactis*, *L.amylolyticus*, *L .gallinarum* and *L. jensenii* | TTCGGATCGTAAAGCTCTGTTG TTGGTGAAGAAGGATAGA | NT | No signal generated |
| LJOHN208 | S-S-Bact-0208-a-S-40 | *Lactobacillus johnsonii* | TTTGAAAGATGGTTCTGCTATC ACTCTTGGATGGACCTGC | FP | Non-specific |
| LJOHN74 | S-S-Bact-0074-a-S-40 | *Lactobacillus johnsonii* | CTAAATGAAACTAGATACAAG CGAGCGGCGGACGGGTGAG | FP | Non-specific |
| SBOV87 | S-St-Bact-0087-a-S-40 | *Streptococcus bovis* | CTTGCTAAAGTTGGAAGAGTGT GCGAACGGGTGAGTAACG | N/T | No signal generated |
| SBOV992 | S-G-Bact-0992-a-S-40 | *Streptococcus bovis* and *S. luteciae* | CCTTACCAGGTCTTGACATCCC GATGCTATTCCTAGAGAT | N/T | No signal generated |

| Probe name | Full name[a] | Target organism(s)[b] | Sequence (5'-3') | Hybridisation with pure culture[c] | Actual specificity |
|---|---|---|---|---|---|
| GAMA785 | S-C-Bact-0785-a-S-40 | Gammaproteobacteria | GGATTAGATACCCTGGTAGTCC ACGCTGTAAACGATGTCG | + | Class |
| PROT1134 | S-P-Bact-1134-a-S-40 | Proteobacteria | ATTAGGTCGGGAACTCAAAGG AGACTGCCAGTGATAAACT | + | Phylum |
| ECOL84 | S-F-Bact-0084-a-S-40 | *Escherichia coli* and *Salmonella* spp. | TTGCTCTTTGCTGACGAGTGGC GGACGGGTGAGTAATGTC | + | Family |
| ECOLI119 | S-S-Bact-0119-a-S-40 | *Escherichia coli* O157:H7 EDL933 | AATGTCTGGGAAACTGCCTGAT GGAGAGGGATAACTACTG | N/T | No signal generated |
| SFLE465 | S-St-Bact-0465-a-S-40 | *Shigella flexneri* | AATACCTTTACTCATTGACGTT ACCCGCAGAAGAAGCACC | FP | Family |
| STYP971 | S-G-Bact-0971-a-S-40 | *Salmonella* Typhimurium and *S. enteritidis* | GCGAAGAACCTTACCTGGTCTT GACATCCACAGAAGAATC | FP | Family |
| ROB592 | S-S-Bact-0592-a-S-38 | *Ruminococcus obeum* | GCGTAGACGGACTGGCAAGTC TGATGTGAAAGGCGGGG | + | Species |
| POSa1055 | S*-Bact-1055-a-S-40 | Most bacteria | ATGGYYGTCGTCAGCTCGTGCC GTGAGGTGTYGGCTTAAG | + | Domain bacteria |
| POSb1055 | S*-Bact-1055-a-S-40 | Most bacteria | ATGGYYGTCGTCAGCTCGTGTC GTGAGATGTYGGGTTAAG | + | Domain bacteria |
| POSc1055 | S*-Bact-1055-a-S-40 | Most bacteria | ATGGYYGTCGTCAGCTCGTGTT GTGAAATGTYGGGTTAAG | + | Domain bacteria |

| Probe name | Full name[a] | Target organism(s)[b] | Sequence (5'-3') | Hybridisation with pure culture[c] | Actual specificity |
|---|---|---|---|---|---|
| SCER1291 | S-P-Euk-S.cere-1291(S. cerevisiae isolate WI1)-a-S-40 | *Saccharomyces cerevisiae* and *S. bayanus* | TGGTGGAGTGATTTGTCTGCTT AATTGCGATAACGAACGA | - | No signal from any labelled DNA recorded |
| TMAR164 | S-St-Bact-0164-a-S-40 | *Thermotoga maritima* | GAAACCCTGGTTAATACCCCAT ACGCTCCATCAACGCAAG | - | No signal from any labelled DNA recorded |

[a] Nomenclature of the oligonucleotide probe according to Alm *et al*. [57].

[b] Target organism(s) of the probe from the BLASTn prediction [17].

[c] Hybridisation with pure culture results: +, positive hybridisation; -, no hybridisation; NT, not tested; FP, false positive
