# Supplementary material for: A short-oligonucleotide microarray that allows improved detection of gastrointestinal tract microbial communities
Source: BMC Microbiol. 2008 Nov 11;8:195. doi: 10.1186/1471-2180-8-195 (PMC2628385; doi:10.1186/1471-2180-8-195)
Supplement: Additional file 3 — Target organisms for 16–21-mer probes and actual tested microarray specificity. Fifty-two short oligonucleotides (16–21-mer) selected for the pilot microarray. Described specificity testing results are for the optimum hybridisation and first washing temperatures of 58°C and 58°C respectively. [file 1471-2180-8-195-S3.pdf]

Additional file 3. Target organisms for 16-21-mer probes and actual tested microarray specificity.

Probe name	Full name[a]	Target organism(s)	Sequence (5'-3')	Predicted probe specificity[m]	Hybridisation with pure culture[n]	Actual specificity
Bacteroides	S-G-Bact-0400-a-S-19	*Bacteroides fragilis*	CAAGTAGCGTGAAGGATGA	*Bacteroides caecae*, *B. eggerthii*, *B. fragilis*, *B. merdae*, *B. ovatus*, *B. thetaiotaomicron*, *B. uniformis*, *B. vulgatus* and *B. stercoris.*	+	Genus
B. fragilis grp[b]	S-G-Bact-0631-a-S-20	*Bacteroides fragilis*	TAAAATTGCAGTTGATACTG	*Bacteroides fragilis* group	+	Species
B. longum grp2[b]	S-S-Bact-0631-a-S-21	*Bacteroides fragilis*	TAAAATTGCAGTTGATACTGT	*Bacteroides fragilis*, *B. thetaiotaomicron*, *B. stercoris*, *B. vulgatus*, *B. caecae*, *B. uniformis*, *B. eggerthii*, *Prevotella zoogleoformans* and *P. heparinolytica*	FP No Bacteroides species bound, but highly specific for *Bifidobacterium longum* and *B. infantis*	Species - The *Bifidobacterium longum and B. infantis* group
B. distasonis 1[c]	S-G-Bact-0165-a-S-18	Bifidobacteria	GGGTGGTAATGCCGGATG	Specificity to a wide range of bifidobacteria	FP	Species *Bacteroides distasonis*
*B. distasonis*2	S-S-Bact-0750-a-S-18	*Bacteroides distasonis*	TGCCAAGCCATTACTGAC	*Bacteroides distasonis*	FP *Bacteroides fragilis*	Genus
BLONG185	S-S-Bact-0185-a-S-18	*Bifidobacterium longum*, *B. breve* and *B. longum* bv. Infantis	TTCCAGTTGATCGCATGGT	*Bifidobacterium longum*, *B. breve* and *B. longum* bv. Infantis	FP	Non-specific
B. longum grp & *B. breve*	S-G-Bact-0277-a-S-18	*Bifidobacterium longum*, *B. breve* and *B. longum* bv. Infantis	CGACGATCCCAGAGATGG	*Bifidobacterium longum* and *B. longum* bv. Infantis	+	Species

Probe name	Full name[a]	Target organism(s)	Sequence (5'-3')	Predicted probe specificity	Hybridisation with pure culture	Actual specificity
B. longum grp3[d]	S-G-Bact-0445-a-S-18	*Bifidobacterium longum* and *B. infantis*	TATCGGGGAGCAAGCGAG	*Bifidobacterium longum*, *B. infantis*, *B. pullorum*, *B. gallinarum*, *B. saeculare* and *B. subtile*	+	Species
B. pseud & catenulatum[b]	S-G-Bact-0177-a-S-16	*Bifidobacterium pseudocatenulatum*	CGGATGCTCCGACTCC	*Bifidobacterium catenulatum* group and *B. thermacidophilum*	+	Species
B. adolescentis[e]	S-G-Bact-0211-a-S-21	*Bifidobacterium adolescentis*	GGAAAGATTCTATCGGTATGG	*Bifidobacterium adolescentis* and *B. ruminantium*	+	Species
B. angulatum[b]	S-S-Bact-0186-a-S-18	*Bifidobacterium angulatum*	CAGTCCATCGCATGGTGG	*Bifidobacterium angulatum*	+	Species
B. bifidum[b]	S-G-Bact-0186-a-S-21	*Bifidobacterium bifidum*	CCACATGATCGCATGTGATTG	*Bifidobacterium bifidum* and *B. cuniculi*	+	Genus, *Bifidobacterium bifidum and B. longum*
B. longum grp1[b]	S-S-Bact-1006-a-S-18	*Bifidobacterium longum* and *B. longum* bv. Infantis	GTTCCCGACGGTCGTAGA	*Bifidobacterium longum*, *B. pseudolongum* and *B. longum* bv. Infantis	+	Species
Entero-bacteriaceae1	S-F-Bact-0183-a-S-18	Enterobacteriaceae	CGTCGCAAGACCAAAGAG	*Escherichia coli* and *Salmonella* Typhimurium	+	Family

Probe name	Full name[a]	Target organism(s)	Sequence (5'-3')	Predicted probe specificity	Hybridisation with pure culture	Actual specificity
Firmicutes1[f]	S-P-Bact-0366-a-S-18	Firmicutes – probe 1	GCAGTAGGGAATCTTCCG	Too many 16S ribosomal gene target matches to define full probe specificity using BLASTn	+	Genus - *Lactobacillus acidophilus* , *L. gasseri* and *L. johnsonii*
Firmicutes2[f]	S-P-Bact-0366-a-S-18	Firmicutes – probe 2	GCAGTAGGGAATCTTCGG	Too many 16S ribosomal gene target matches to define full probe specificity using BLASTn	+	Genus - *Enterococcus faecium*
Firmicutes3[f]	S-P-Bact-0366-a-S-18	Firmicutes – probe 3	GCAGTAGGGAATCTTCCA	Too many 16S ribosomal gene target matches to define full probe specificity using BLASTn	+	Genus - *Lactobacillus acidophilus* , *L. gasseri* and *L. johnsonii*
L. johnsonii [g]	S-S-Bact-0052-a-S-23	*Lactobacillus johnsonii*	GATGATTTTAGTTCTTGCACTA A	*Lactobacillus johnsonii*	-	N/A
L. casei [h]	S-S-Bact-0092-a-S-20	*Lactobacillus casei*	TGCACTGAGATTCGACTTAA	*Lactobacillus casei*	-	N/A
Enterococcus genus	S-G-Bact-1262-a-S-16	*Enterococcus faecium*	AGTCGCGAGGCTAAGC	Specificity to a wide range of the *Enterococcus* genus	+	*Enterococcus faecium*
E. faecium	S-G-Bact-0097-a-S-18	*Enterococcus faecium*	CGGAAAAAGAGGAGTGGC	*Enterococcus faecium*, *E. canis*, *E. hirae*, *E. villorum*, *E. azikeevi* and *E. durans*	+	*Enterococcus faecium*
R. obeum [i]	S-S-Bact-1455-a-S-20	*Ruminococcus obeum*	ACCTAACTGCAAAGAAGGAG	*Ruminococcus obeum*	NT	No signals from other labelled DNA recorded

Probe name	Full name[a]	Target organism(s)	Sequence (5'-3')	Predicted probe specificity	Hybridisation with pure culture	Actual specificity
Ccluster XIVab[j]	S-*-Erec-0482-a-A-19	*Clostridium coccoides-Eubacterium rectale* group	GCT TCT TAG TCA RGT ACC G	*Clostridium coccoides-Eubacterium rectale* group	NT	No signals from other labelled DNA recorded
C. leptum grp[k]	S-G-Clept-1240-a-A-18	*Clostridium leptum*	GTTTTRTCAACGGCAGTC	*Clostridium leptum* subgroup	+	Target group
*C. leptum*1[l]	S-S-Bact-0205-a-S-22	*Clostridium leptum*	ATAGGTTGATCAAAGGAGCAAT	*Clostridium leptum*	+	Species
*C. leptum*2	S-S-Bact-0441-a-S-21	*Clostridium leptum*	ACCTCTGTTCTTAGTGACGAT	*Clostridium leptum*	+	Species
Salmonella genus (SAL455)	S-F-Bact-0455-a-S-20	*Salmonella* Typhimurium	TGTTGTGGTTAATAACCGCA	Specificity to a wide range of the *Salmonella* genus	+	Genus
Universal 1	S-D-Bact-0338-a-A-18	Superkingdom bacteria	GCTGCCTCCCGTAGGAGT	Most bacteria	+	Domain bacteria
Thermus Thermoph1	S-G-Bact-0200-a-S-20	*Thermus thermophilus*	GGTGTGTCCAAAGGGC	*Thermus thermophilus*	+	Species
Thermus Thermoph2	S-G-Bact-0158-a-S-18	*Thermus thermophilus*	GGGAAACTCGGGCTAATC	Thermus genus	+	Target species
Thermus Thermoph3	S-G-Bact-1012-a-S-16	*Thermus thermophilus*	CGGGTGAAAGCCTGGG	*Thermus thermophilus*	+	Species
BLON135a	S-G-Bact-0135-a-S-19	*Bifidobacterium longum*, *B. infantis* and *B. adolescentis*	GCCCCATACACCGGAATAG	Probe used for melting curve profile only. It was not applied to determine bacterial presence	+	N/A

Probe name	Full name[a]	Target organism(s)	Sequence (5'-3')	Predicted probe specificity	Hybridisation with pure culture	Actual specificity
BLON135b	S-G-Bact-0135-a-S-19	*Bifidobacterium animalis*	GCCCCATGCACCGGAATAG	Probe used for melting curve profile only. It was not applied to determine bacterial presence	+	N/A
BLON135c	S-G-Bact-0135-a-S-19	*Bifidobacterium bifidum*	GCCCCAGACTCCGGAATAG	Probe used for melting curve profile only. It was not applied to determine bacterial presence	+	N/A
Negative control1	N/A	None	AGTCAGTCAGTCAGTCAGTC	None	-	-
Negative control2	N/A	None	AAGGTTCCAAGGTTCCAAGG	None	-	-
Negative control3	N/A	None	GGAAACCCTTTGGGAAACCC	None	-	-
Negative control4	N/A	None	GGAAACCTGTAGCCCTTTCC	None	-	-
Negative control5	N/A	None	GGGTTTCACAAGGTAAGAATG TG	None	-	-

[a] Nomenclature of the oligonucleotide probe according to Alm et al. [57]; [b] Probe designed by Matsuki et al. [58]; [c] Probe designed by Kok et al. [59]; [d] Probe designed by Roy et al. [53]; [e] Probe designed by Wang et al. [60], [f] Probe designed by Meier et al. [61], [g] Probe designed by Ventura et al., [62]; [h] Probe designed by Ward and Timmins, [63]; [i] Probe designed by Wang et al. [64]; [j] Probe designed by Franks et al. [1], [k] Probe designed by Sghir et al. [65]; [l] Probe designed by Wang et al. [66].

[m]Predicted probe specificity indicates the proposed specificity using BLASTn [17] during February 2006.

[n]Hybridisation with pure culture results: +, positive hybridisation; -, no hybridisation; NT, not tested; FP, false positive
