# Supplementary material for: A short-oligonucleotide microarray that allows improved detection of gastrointestinal tract microbial communities
Source: BMC Microbiol. 2008 Nov 11;8:195. doi: 10.1186/1471-2180-8-195 (PMC2628385; doi:10.1186/1471-2180-8-195)
Supplement: Additional file 4 — Oligonucleotide probes, mainly between 16- and 21-mer in length, used for the expanded community microarray. Unique oligonucleotide probes mainly 16–21-mer in length designed for the short community microarray. Complete list of 230 short oligonucleotide probes used in this study including the predicted specificity. [file 1471-2180-8-195-S4.pdf]

Additional file 4 - Unique oligonucleotide probes mainly 16-21-mer in length designed for the short community microarray. The total number of probes designed were 230.

Probe name	T_m (NN)	Probe length	Probe Sequence (5'-3')*	Predicted probe specificity and nucleotide differences to other bacterial 16S ribosomal gene sequences	Reference	Notes
A. laidlawii 1	49.9	18	TGTGGTGTAAGTGCAGTG	*Acholeplasma laidlawii* and *Mesoplasma pleciae*. All other seqences had at least 2 nt differences and were eukaryotic.	This study	
A. laidlawii 2	49.5	19	CGGAGGCTWACAGATGTAC	*Acholeplasma laidlawii* and *Mesoplasma pleciae*. 1 nt difference to *Acholeplasma palmae* and remaining sequences had at least 3 nt differences, all of which were eukaryotic.	This study	
A.laidlawii 3	49.7	17	ACGCTGTGAGGCTATGA	*Acholeplasma laidlawii*, no other similar sequences with homology to this probe.	This study	
A.laidlawii 4	43.1	16	GGATGTGTGCATGAAA	*Acholeplasma laidlawii*, no other similar sequences with homology to this probe.	This study	
A. fermentans	52.0	24	GCACCGATCTATAATACATTTTGG	*Acidaminococcus fermentans*, no other similar sequences with homology to this probe.	This study	
Actinobacterium genus	49.9	18	ACTCGCAGAATAAGCACC	Specific for all *Acintobacterium*. Also specific to *Achromobacter xylosoxidans*, *Paenibacillus* spp., *Pseudomonas aeruginosa* (all 3 from oily sludge), *P. citronellolis* (unknown source), *Derxia gummosa*, *Alkanindiges hongkongensis* and one eukaryotic sequence. 1 nt difference from an *Acinobacter* spp. and all remaining sequences had at least 2 nt differences to this probe.	This study	
Alphaproteobacteria	52.0	18	GGTAAGGTTCTGCGCGTT	Alphaproteobacteria (except Rickettsiales).	Neef *et al.* (1997) (probeBase)	84.7% total coverage
Alpha-Deltaproteobacteria	52-54	17	CGTTCGYTCTGAGCCAG	Alphaproteobacteria, some Deltaproteobacteria and Spirochaetes.	Manz *et al.* (1992) (probeBase)	84.7% total coverage
A. burkinabensis	51.0	22	TTTCGCATGGAGATGATA	*Anaerovibrio burkinabensis*. Next nearest bacterial sequence homology has 4 nt difference to this probe.	This study	
B. alcalophilus	49.3	17	CCGTTCGAATAGGTCGG	*Bacillus alcalophilus*, six *Bacillus* spp., seven uncultured microorganisms, *Cerasibacillus quisquiliarum* (non gut source) and *Brevibacillus* spp. No further sequence homologies	This study	
B. distasonis 1#	50.0	18	GGGTGGTAATGCCGGATG	This probe has specificity to a wide range of Bifidobacteria but none bound in the pure culture tests. However it did have specificity to *Bacteroides distasonis* which was complemented by the B. distasonis2 probe.	Matsuki *et al.* (2002)	

B. distasonis 2#	50.0	18	TGCCAAGCCATTACTGAC	*Bacteroides distasonis* and two uncultured bacteria from the human gut. 2 nt difference to two uncultured deep sea bacteria, *Mycobacterium leprae, Yersinia pestis* and *Streptococcus pneumoniae*. At least 3 nt differences to all remaining sequences, most of which were eukaryotic.	This study	
B. fragilis grp#	45.0	20	CAGTATCAACTGCAATTTTA	*Bacteroides fragilis, B. thetaiotaomicron*, *B. stercoris, B. vulgatus*, *B. caecae*, *B. uniformis, B. eggerthii*, *Prevotella zoogleoformans* and *P. heparinolytica*. All other sequence hits had at least 2 nt difference from this probe and many were uncultured.	Matsuki *et al*. (2002).	The first 5' nucleotide was removed from the primer to cover all *B. fragilis* strains in the Genbank database.
B. longum grp2#	46.0	21	TAAAATTGCAGTTGATACTGT	*Bacteroides fragilis*, twelve uncultured bacteria from human, equine, waste water, and wet wood sources. 1 nt difference to *Bacteroides thetaiotaomicron*, B. stercoris, *B. vulgatus*, *B. caecae*, *B. uniformis*, *B. eggerthii*, *Prevotella zoogleoformans*, *P. heparinolytica* and many unidentifed bacteria from human gut sources. 2 nt difference or more to all remaining sequences.	Matsuki *et al.* (2002)	
Bacteroides#	50.0	19	CAAGTAGCGTGAAGGATGA	*Bacteroides caecae*, *B. eggerthii*, *B. fragalis*, *B. merdae*, *B. ovatus*, *B. thetaiotaomicron*, *B. uniformis*, *B. vulgatus*, *B. stercoris*. Also covers one strain of *Prevotella heparinolytica* and many uncultured bacteria from human, chicken, rumen, swine pit and equine sources. 1 nt difference to uncultured faecal bacteria, 2 nt difference to two further uncultured faecal bacteria and 3 nt difference to eighteen others including *Salmonella* Typhi and *Enterococcus faecium*.	This study	
B. merdae	50.0	17	TGGCGACAGGATGCTAA	*Bacteroides merdae*, *Bacteroides acidofaciens* and *Bacteroides eggerthii*. 1 nt difference to several eukaryotic sequences and 2 nt difference to *Prevotella tannerae* and *Bacteroides ovatus.*	This study	
B. ovatus	50.2	19	TTACTGACACTGATGCTCG	*Bacteroides ovatus* and one uncultured *Bacteroides* spp. 2 nt difference to *Bacteroides fragilis,* B. thetaiotaomicron, *B. acidofaciens*, *Yersinia pestis* and fifteen uncultured bacteria from the human gut.	This study	
B. putredinis 1	50.0	16	GCAGGATGACGGCTCT	*Bacteroides putredinis,* three *Bacteroides* spp. and six uncultured bacteria from human and rumen GI tracts. 1 nt difference to *Salmonella* enterica, *Brucella melitensis* and *B. suis*. All remaining similar sequences were eukaryotic	This study	

B. putredinis 2	49.9	20	ATTCCGATGGTTAGAGATGG	*Bacteroides putredinis* and two uncultured bacteria from the human gut. All remaining similar sequences were eukaryotic and had 5 nt difference to this probe.	This study	
B. putredinis 3	50.0	17	AAGTTCGTGACCGCAAG	*Bacteroides putredinis* and twenty-one other unknowns from rumen, human and equine GI tract sources. 2 nt difference to the only other bacteria with a sequence similarity to this probe - *Mycobacterium avium.*	This study	
B. putredinis 4	50.1	19	TACCGTTAGTTGCCATCAG	*Bacteroides putredinis* and thriteen other unknown GI tract bacteria from human and mice. 1 nt difference to *Desulfotomaculum alkaliphilum* and two uncultured bacteria from mice GI tracts, 2 nt difference to sixteen other bacteria whose enumerated source in included the rumen, termite and horse gut. 3 nt difference to many other bacteria.	This study	
B. splanchnicus	49.9	20	TCCTATTAGTTAGTTGGCGG	*Bacteroides splanchnicus* , two planctomycetales clones, a single uncultured bacteria and a *Eubacterium* clone. 1 nt difference to a second plantomycetes clone and all remaining similar bacterial sequences had 3 nt difference.	This study	
B. stercoris	50.1	19	TGAATCGGAAACGGTTCTT	*Bacteroides stercoris* . 1 nt difference to two uncultured bacteria from the human gut. All remaining similar sequences were eukaryotic and had 2 to 4 nt differences.	This study	
B. thetaiotaomicron & *B. ovatus*	51.0	23	GGTACATACAAAATTCCACACGT	*Bacteroides thetaiotaomicron* , *B. ovatus* and fifteen uncultured or *Bacteroides* spp. from human and Crohn's disease patients. 1 nt difference to *B. acidofaciens* , *B. caccae* and several other uncultured bacteria from the human gut. The next similar sequences had 6 nt difference to this probe.	Wang *et al.* (1996)	
B. thetaiotaomicron	52.0	20	GGCAGCATTTCAGTTTGCTT	*Bacteroides thetaiotaomicron* and four uncultured bacteria from the human gut. 2 nt difference or more and all remaining sequences which were eukaryotic.	Wang *et al.* (1996)	The 3' G of the primer was removed to bring the NN temperature to within the microarray probe boundaries.
B. vulgatus 1	49.9	17	CGCAAGGCATCTGTGAA	*Bacteroides vulgatus* and thirty-six uncultured microorganisms. 1 nt difference to *Methanosarcina mazei* and 2 nt difference or more all other sequences which were eukaryotic.	This study	
B. vulgatus 2	50.1	19	CTACTCTTGGACAGCCTTC	*Bacteroides vulgatus* and five uncultured bacteria from the human gut. 2 nt difference to twenty-five uncultured bacteria from the human gut and 4 nt difference to remaining sequences which were all eukaryotic.	This study	

B. adolescentis #	49.0	21	GGAAAGATTCTATCGGTATGG	*Bifidobacterium adolescentis* , *B. ruminatum* and five unknown bacteria. 1 nt difference to two *Bifidobacterium* spp. from human faeces and two *B. angulatum* strains. 5 nt difference to *B. bifidium* and all remaining similar sequences had 6 nt differences and belonged to eukaryotes.	Matsuki *et al.* (2002).	
B. angulatum #	52.0	18	CAGTCCATCGCATGGTGG	*Bifidobacterium angulatum* . 2 nt difference to the next nearest similar sequences which were all *Streptomyctes*	Matsuki *et al.* (2002)	The 3' T was removed so the NN temperature was within the temperature range of microarray probes.
B. bifidum #	52.0	20	CCACATGATCGCATGTGATTG	*Bifidobacterium bifidum* , *B. cunniculi* and four uncultured bacteria from the human gut. 4 nt difference or more to all remaining sequences which were eukaryotic.	Matsuki *et al.* (2002)	The 3' G was removed so the NN temperature was within the temperature range required for the microarray probes
B. breve	51.0	18	CCGGATGCTCCATCACAC	*Bifidobacterium breve.* 3 nt difference to any other sequences which were all were eukaryotic.	Matsuki *et al.* (2002)	
B. longum grp & *B. breve* #	52.0	18	CGACGATCCCAGAGATGG	*Bifidobacterium infantis* , *B. breve* , *B. indicum* , four uncultured bacteria from the human gut and one *B. longum* strain. 2 nt difference to all remaining sequences which were all eukaryotic.	Matsuki *et al.* (2002)	The 3' G was removed from the end of the primer to bring it into the NN temperature range of the microarray.
Bifidobacterium longum general group#	51.0	19	TTCCAGTTGATCGCATGGT	*Bifidobacterium longum* , *B. infantis* , *B. suis* , *B. pseudolongum* . The only other similar sequence were from eukaryotes. Note: This probe is non-specific	Matsuki *et al.* (2002)	
B.longum grp1#	52.0	18	GTTCCCGACGGTCGTAGA	*Bifidobacterium longum* , *B. pseudolongum* , *B. infantis* and fifteen uncultured bacteria all from human/infants faeces. 1 nt difference to unidentified *Bifidobacterium* spp.. 3 nt difference from *B. suis* , *B. dentium* and all remaining similar sequences were eukaryotic.	Matsuki *et al,* (2002)	The 3' G of the primer was removed to bring the NN temperature to within the range set for the microarray.
B. longum grp3#	54.0	18	TATCGGGGAGCAAGCGAG	*Bifidobacterium longum* , *B. infantis* , *B. pulluroum* , *B. gallinarum* , *B. saeculare* and *B. subtile* . 2 nt to the next nearest sequences which were eukaryotic	Roy *et al* . (1996)	Removal of the 3' CT brought the NN temperature down to as close to the microarray boundaries as possible without compromising specificity.
B. pseud & *catenulatum* #	50.0	16	CGGATGCTCCGACTCC	*Bifidobacterium pseudocatanulatum* , *B. catanulatum* , *B. thermacidophilum* and ten uncultured bacteria from humans, 1 nt to next similar sequences which were all eukaryotic.	Matsuki *et al.* (2002)	Removal of the 3' T brought the NN temperature to within the range required for the microarray.

B. crossotus	50.1	18	CGGCATCACAAGTCAGAA	*Butyrivibrio crossotus.* 1 nt difference to five uncultured bacteria and *Lachnospira pectinoschiza* from the pig GI tract. All remaining similar sequences had at least 3 nt difference to this probe.	This study	
B. fibrisolvens	48.0	18	GACCTCGTAAGAGGGGAT	*Butyrivibrio fibrisolvens* and one uncultured rumen bacteria. 2 nt difference to the only other bacterial sequences.	This study	
C. jejuni	46.1	19	AGTTGAGTAGGGAAAGTTT	*Campylobacter jejuni* and *C. coli* . 3 nt difference to all remaining similar sequences which all were eukaryotic.	This study	
C. coli & jejuni	48.0	23	ATCTAATGGCTTAACCATTAAAC	*Campylobacter coli* , *C. helveticus* , *C. hyointestinalis* , *C. jejuni* and *C. upsaliensis* . 2 nt difference to the next nearest sequences, all of which were eukaryotic.	Inglis and Kalischuk (2003)	
Campylobacter	50.0	19	GGATGACACTTTTCGGAGC	The *Campylobacter* genus. 2 nt difference to *Streptococcus mutans* and 3 nt to an uncultured *Eubacterium* . All remaining similar sequences were eukaryotic.	Inglis *et al.* (2003)	
Carnobacteria	47.0	18	GAATCATGCGATTCCTGA	*Carnobacterium gallinarum, C. piscicola* , *C. maltaromaticum* and one uncultured *Clostridium* spp. from a non-gut source. 3 nt difference to next nearest bacterial sequences.	Barakat *et al.* (2000)	2 nts were removed from the 3' end of the primer to bring the NN temperature range of the probe within the range of the microarray
C. butyricium	52.0	20	TACCGCATGGTACAGCAATT	*Clostridium butyricium* and thirteen uncultured bacteria from pig GI tracts, waste water and human guts. 1 nt difference to nineteen uncultured bacteria from rumen, human and pig GI tracts. All remaining similar seqences had 5 nt difference to this probe and were eukaryotic.	Kikuchi *et al.* (2002)	
C. aminovalericum	47.7	19	GTTCAACTTCGTTSAACTC	*Clostridium aminovalericum.* Specific by 4 nt to any other organism. To cover both *C. aminovalericum* (M23929 and X73436) at position 14 where a C is present in one sequence and a G in the other, the probe was made degenerate.	This study	
C. bifermentans	52.0	17	CAAGTCGAGCGATCTCT	*Clostridium bifermentans* , five uncultured bacteria from swine pits or deepsea hydrothermal vents and a single *C. paraputrificum* . 1 nt difference to two uncultured bacteria and C. *hiranonis.* 2 nt difference to *C. lituseburense* and a single uncultured bacteria from cow faeces.	Kikuchi *et al.* (2002)	
C. celatum	50.2	21	ACGATGAATACTATGAGCACT	*Clostridium celatum* . 5 nt difference to *Bacillus cereus* and all remaining similar sequences had 6 nt difference.	This study	

C. clostridiformes 1	49.0	16	CACCGAAGGCTTTGCC	*Clostridium clostridiformes* and four uncultured bacteria from the human GI tract, sewage and rumen. 1 nt difference to *Pseudomonas putida* , *Bradyrhizobium japonicum and Pyrococcus abyssi* (all from non-gut sources). All remaining similar sequences were eukaryotic.	Kikuchi *et al.* (2002)	
C. clostridiformes 2	52.0	23	GAAGTTTTCGGATGGAATCTTGA	*Clostridium clostridiformes* and one uncultured bacteria from the human gut. Note: This probe only covered half the strains from this species when clustalled. 2 nt difference to uncultured bacteria from activated sludge and the next most similar sequences were divergent by 4 nt.	Kikuchi *et al.* (2002)	
CclusterI	43-52	21	CARGRGATGTCAAGYCYAGGT	Too many 16S ribosomal gene target matches to define full probe specificity using BLASTn	Van Dyke and McCarthy (2002)	
CclusterIII	38-51	19	GRCAGTATDCTGACCTRCC	Too many 16S ribosomal gene target matches to define full probe specificity using BLASTn	Van Dyke and McCarthy (2002)	
CclusterIV	43.0	17	TTACTGGGTGTAAAGGG	Too many 16S ribosomal gene target matches to define full probe specificity using BLASTn	Van Dyke and McCarthy (2002)	
CclusterXIVab#	47.0-50.0	19	CGGTACYTGACTAAGAAGC	Too many 16S ribosomal gene target matches to define full probe specificity using BLASTn	Van Dyke and McCarthy (2002)	
C. coccoides	49.0	19	GCTTCTTAGTCARGTACCG	*C. coccoides* and many uncultured bacteria	Franks *et al* . (1998)	
C. cocleatum	50.0	19	CTCGGATGTCATTTCCTCC	*Clostridium cocleatum* and 2 nt difference to the next most similar sequences which were all eukaryotic.	Kikuchi *et al.* (2002)	
C. difficile	50.0	21	CTACAATCCGAACTGAGAGTA	*Clostridium difficile* , four uncultured bacteria from hydrothermal vents and *Alvinella pompejana* . 1 nt difference to twenty-three strains including five *Bacillus* spp. and five uncultured bacteria. Next most similar sequences had 2 nt differences and many were from *Helicobacter* spp. and *Enterococcus* spp.	Kikuchi *et al.* (2002)	Only five examples of *C. difficile* were present in the database
C. innocuum	50.0	17	GTCGCTGCTCTTTGTGG	*Clostridium innocuum* . 2 nt difference to the next nearest similar prokaryotic sequence which included *Eubacterium cylindroides* and many eukaryotes.	Kikuchi *et al.* (2002)	
C. leptum 1#	51.0	21	ACCTCTGTTCTTAGTGACGAT	*Clostridium leptum* . 1 nt difference to two uncultured bacteria from non-gut sources and eukaryotes. All remaining similar seqences had 5 nt or more divergence.	Wang *et al.* (1994)	
C. leptum 2#	51.0	22	ATAGGTTGATCAAAGGAGCAAT	*Clostridium leptum* . 4 nt difference to an uncultured bacterium from soil and all remaining sequences had 5 nt divergence.	Wang *et al.* (1994)	
C. leptum grp#	45-46	18	GTTTTRTCAACGGCAGTC	Too many 16S ribosomal gene target matches to define full probe specificity using BLASTn	Sghir *et al.* (2000)	

C. nexile	49 - 53	18	RCCTGYGAGGGKAAGCAA	*Clostridium nexile* , four bacteria from non-gut sources, a single uncultured bacteria from the rumen of a yak and five uncultured bacteria from the human gut. 2 nt or greater sequence divergence from any further prokaryotic sequences.	Kikuchi *et al.* (2002)	After clustaling, the primer was found only to cover two *C. nexile* sequences in the NCBI database. It needed three variable nucleotides to added for coverage of the remaining strains.
C. paraputrificum	49.0	22	CCTGAATTACCATGTAATGTGG	*Clostridium paraputrificum* and one uncultured sequence from a human new-born. 5 nt difference to any other bacteria.	Kikuchi *et al.* (2002)	
C. perfringens 1	48.0	20	AGATGGCATCATCATTCAAC	*Clostridium perfringens* and eleven uncultured sequences from human, pigs and rat GI tracts plus from unidentified sources. 3 nt difference to the next most similar bacteria which was an unculured from the human gut.	Kikuchi *et al.* (2002)	
C. perfringens 2	48.0	18	GCAAGGGATGTCAAGTGT	*Clostridium perfringens* and nine uncultured bacteria from human and pig GI tracts. 2 nt difference to several Clostridia from the gut human, the most common match from *Clostridium botulinum* .	Kikuchi *et al.* (2002)	
C. perfringens 3	48.0	20	AGATGGCATCATCATTCAAC	*Clostridium perfringens* and eleven uncultured from human, pig and rat GI tracts. 3 nt difference to a few uncultured bacteria from the human gut.	Kikuchi *et al.* (2002)	
C. perfringens 4	45.4	18	ATGGCATCATCATTCAAC	Only one strain of *Clostridium perfringens* and 10 uncultured bacteria from a variety of sources. All other *C. perfringens* strains had 2 nt divergence to this probe	This study	This probe was not added to the microarray because the NN temperature was below the range required and there were already three other *C. perfringens* probes.
C. proteolyticum	50.0	20	CCTTGTTGTTAGTTGCTACC	*Clostridium proteolyticum* , *C. histolyticum* , *Clostridium limosum* and five unknown bacteria from sewage. 1 internal nt difference to a large number of Clostridia. 2 nt differences to *Flavobacterium psychrophilum* and unknown bacteria.	This study	
C. ramosum 1	51.0	21	GTGACCGTATTAAAAGTGCCT	*Clostridium ramosum* and 5 uncultured bacteria (only 2 from a gut source). 2 nt difference to *C. cocleatum* and *C. spiroforme* . Remaining sequence matches had 6 nt or more divergence.	Kikuchi *et al.* (2002)	
Clostridium ramosum	49.0	17	TACCGTCACTCGGCTAC	*Clostridium ramosum* and four uncultured bacteria, two of which were from gut sources. 1 nt difference to eukaryotic sequences and 2 nt divergence to two uncultured bacteria from non-gut sources. No other sequence matches.	Kikuchi *et al.* (2002)	

C. sordellii	51.0	16	GTCGAGCGACCTTCGG	*Clostridium sordellii* and *Rhodovulum* spp. (phototrophic bacteria). 1 nt difference to *Streptococcus mutans* (human oral cavity) and all remaining sequences did not originate from gut sources.	Kikuchi *et al.* (2002)	This study removed the G from the 5' position of the probe to bring the NN melting temperature into range.
C. leptum sub-group	53-57	18	TAAAGCCCAGYAGGCCGC	*Clostridium sporosphaeroides* , *Ruminococcus bromii* , *C. leptum* and one unknown bacteria from rumen fluid. Too many 16S ribosomal gene target matches from uncultured bacteria to define full probe specificity using BLASTn.	Harmsen *et al.* (2002)	
C. symbiosum	50.0	19	GCGACTTACTGGACGATAA	*Clostridium symbiosum* and three uncultured bacteria. The probe was specific by 1 nt to ten unknown bacteria and 2 nt to further unknown bacteria.	This study	
C. viride	50.7	19	CAAGTTTTCGGACAAGGGA	*Clostridium viride* and three unidentified bacteria. 2 nt difference to one uncultured bacteria and 3 nt to further uncultured bacteria.	This study	
E. aerofaciens	50.0	19	CTTTCAGCAGGGAAGAGTC	*Collinsella/Eubacterium aerofaciens, Coriobacterium* spp. (both from human faeces) and three unknown bacteria from human faeces. 2 nt difference to all other sequences which were eukaryotic.	Tajima *et al.* (2000)	
C. catenaformis 1	50.3	16	TCGCATGACCGCTTCA	*Coprobacillus catenaformis* . 1 nt difference to *Bradyrhizobium japonicum* , *Mesorhizobium loti* and *Geobacter sulfurreducens* . No other sequences were similar.	This study	
C. catenaformis 2	49.7	20	AATAYCCGTATGGATAGCAG	*Coprobacillus catenaformis* . All remaining bacterial sequences had 5 nt difference from this probe.	This study	
C. eutactus 1	50.0	18	TCCTGATGACGGTTCCTT	*Coprococcus eutactus* and three other uncultured bacteria all from cow/human guts. 3 nt difference to *Salmonella* enterica, *S.* Typhimurium, *S.* enteritidis and eukaryotic sequences.	This study	
C. eutactus 2	50.0	19	TACTGGACTGCTACTGACA	*Coprococcus eutactus* , *Oscillatoria terebriformis* and three uncultured bacteria from the human gut. 2 nt difference to uncultured bacteria and 3 nt divergence to all remaining sequences.	This study	The only example in the database of this species when the probe was designed.
Corynebacterium	48.0	17	TTCACACCACCGTAGGG	*Corynebacterium glutamicum* , *Brevibacterium taipei* , *B. seonmiso* , *B. saccharolyticum* , *B. glutamigenes* (All proposed to belong to the *Corynebacterium* group), *Rhodococcus globerulus* , *Pyrococcus abyssi* (Archaea) and five uncultured bacteria. 1 nt difference to all remaining sequences which were eukaryotic.	This study	
C. fermentans 1	49.7	20	TAGGCTGCATAGAGTAGAGA	*Cytophaga fermentans* . 5 nt difference to the next nearest bacterial sequence.	This study	

C. fermentans 2	50.0	23	CCGCATGGTAAATTATATAAAGC	*Cytophaga fermentans* . 4 and 5 nt difference to next similar sequences, all of which were eukaryotic. 6 nt difference to the next nearest bacterial sequence.	This study	
C. fermentans 3	50.5	23	CCGCATGGTAAATTATATAAAGC	*Cytophaga fermentans* . 4 and 5 nt difference to the next most similar sequences.	This study	
Cytophaga-Flavobacterium grp	49.0	18	TGGTCCGTGTCTCAGTAC	Cytophaga-Flavobacterium-group of the Bacteroidetes	Manz *et al.* (1992) (probeBase)	90.5% total coverage
Cytophagales-Bacteroidales grp	47.0	18	CCAATGTGGGGGACCTT	Cytophagales - Bacteroidales-group of the Bacteroidetes	Manz *et al.* (1992) (probeBase)	90.5% total coverage
Cytophaga-Bacteroidetes1	48.0	18	TCCTCTCAGAACCCCTAC	Cytophagales - Groups of the Bacteroidetes phylum	Weller *et al.* (2000) (probeBase)	90.5% total coverage
Cytophaga-Bacteroidetes2	49.0	18	CCTTGGTAAGGTTCCTCG	Cytophagales - Groups of the Bacteroidetes phylum	Weller *et al.* (2000) (probeBase)	90.5% total coverage
Cytophaga-Bacteroidetes3	44 (ProbeBase described NN temp as 46°C)	18	GGACCCTTTAAACCCAAT	Cytophagales - Groups of the Bacteroidetes phylum	Weller *et al.* (2000) (probeBase)	90.5% total coverage
Cytophaga-Bacteroidetes4	56 (ProbeBase described NN temp as 53°C)	18	AGCTGCCTTCGCAATCGG	Cytophagales - Groups of the Bacteroidetes phylum	Weller *et al.* (2000) (probeBase)	90.5% total coverage
D. piger 1	50.1	16	GGACTTCGGTCCCGAG	*Desulfovibrio piger* , an uncultured *Acidobacterium* and uncultured bacteria from the termite gut. 1 nt difference to *Caulobacter crescentus, Pseudomonas aeruginosa* and eukaryotic sequences. No other similaraties to other sequences.	This study	
D. piger 2	47.7	20	CTCATGATGAACTTTGTGAG	*Desulfovibrio piger* , *D. oviles* and two unknown bacteria from the termite gut. Next nearest similar bacterial sequence has 5 nt difference.	This study	
D. piger 3	50.0	19	GGAACCCTCCCGAAAATGAG	*Desulfovibrio piger* . There were 3 nt differences to several other *Desulfovibrio* s.	K. Tajima *et al.* (2001)	
D. piger 4	51°C	23	GGGTGTTCTAATCATCATCCTAC	*Desulfovibrio piger* . 1 nt difference to an uncultured bacterium, and 2 nt to other D.piger. Not all D. piger will bind to this probe	This study	Clustal as the other *D. piger* (gi|174333|gb|M34404.1|DSLRR165) only has 17 bp recognition while this primers seq is 23 bp long
Enterobacteriaceae1[#]	52.0	18	CGTCGCAAGACCAAAGAG	*Escherichia coli* , *Enterobacter* spp., *Klebsiella* spp., *Salmonella* spp., *Pantoea* spp., *Citrobacter* spp., *Serratia marcescens* , *Shigella* spp., *Pectobacterium* , *Leclercia adecarboxylata* and *Hafnia alvei* . All are members of the Enterobacteriaceae	Friedrich *et al.* (1999)	

Enterobacteriaceae2	49.6	19	CAGAGATGAGAATGTGCCT	General *Escherichia coli* , *Salmonella* and *Shigella* probe. 1 nt difference to two *E. coli.* 2 nt difference to another two *E. coli* strains and remaining similar sequences were divergent by 3 nt or more.	This study	
Enterococcus genus[#]	52.0	16	AGTCGCGAGGCTAAGC	*Enterococcus faecium* , *E. faecalis* , *E. avium* , *E. hermanniensis* , *E. gallinarum* , *E. sulfureus* , *E. saccharolyticus* , *E. flavescens* , *E. dispar* , *E. pseudoavium* , *E. raffinosus* , *E. mundtii* , *E. malodoratus* , *E. hirae* , *E. malodoratus* , *E. casseliflavus* , *E. mundtii* , *E. villorum* , *E. asini* , *E. pallens* , *E. gilvus* , *E. azikeevi* , *E. malodoratus* . The only other strains with sequence homology were many Wolbachia (they infect invertebrates only), *Bacillus carboniphilus* (unknown origin) and *Carnobacterium alterfunditum* (antartic origin) and twenty-three unknown bacteria from the hindgut of the humus-feeding larva. 1 nt difference to all other sequences which were eukaryotic. No other prokaryotic sequences match this probe.	Netherwood *et al.* (1999)	The 3' T was removed from the primer to bring the NN temperature into range for this experiment.
E. cecorum	50.0	17	CAATGCGGAGGAAGGTG	*Enterococcus cecorum* , *E. columbae* , *Geobacillus jurassicus* , *Dialister* spp., *Xylella fastidiosa* , *Peptococcus niger* , five unidentified bacteria from human, pig or chicken GI tracts and more than fifty other uncultured prokaryotic strains.	This study	
E. faecalis	50.6	16	CTCCCGAGTGCTTGCA	*Enterococcus faecalis* and three unknown bacteria from swine pit manure. 1 nt difference to *Listeria innocua* and all remaining similar sequences were eukaryotic.	This study	
E. faecium[#]	50.0	18	GCCACTCCTCTTTTTCCG	*Enterococcus faecium* , *E. canis* , *E. hirae* , *E. villorum* , *E. azikeevi* and *E. durans* . 1 and 2 nt differences only to eukaryotic sequences. The only other prokaryotic sequence with similarities to this probe was *Yersinia pestis* with 3 nt divergence to this probe.	Behr *et al.* (2000) (probeBase)	
E. coli	54.0	19	CACCGTAGTGCCTCGTCAT	*Escherichia coli* , *Salmonella* Typhimurium LT2, *Shigella flexneri* , *Citrobacter freundii,* *Salmonella* enterica and *Klebseilla pneumoniae* . 2 nt difference to more *Salmonella* and *Klebsiella* strains.	Marteau *et al.* (2001)	The final 2 nts were removed to bring the NN temperature in the range of that selected for the microarray.
E. coli O157	49.0	24	CCATAATCATTTTATTTAGAGGGA	*Escherichia coli* O157. 5 nt difference to all other sequences which were eukaryotic.	Meng *et al.* (1996)	
E. biforme 1	50.0	16	CGAAGGCAGGACCGAT	*Eubacterium biforme* and fifteen uncultured bacterial sequences from equine, human, sludge and pig sources. 1 nt difference to *Bordetella pertussis* but no other similar sequence matches.	This study	

E. biforme 2	50.0	16	ACAGAAGAGGACGGCG	*Eubacterium biforme* and twenty-two uncultured bacteria, mostly from human and pig GI tracts. 1 nt difference or more to all remaining sequences which were eukaryotic.	This study	
E. biforme 3	50.0	19	CGGATAGGTAGCAGACAAG	*Eubacterium biforme* and twenty-four unidentified sequences including from pig and human GI tracts. 3 nt to two strains from the gut and the remaining similar sequences were all eukaryotic.	This study	
E. biforme 4	50.1	17	CGTCTGGTCTGTTGCTG	*Eubacterium biforme* and three unidentified bacteria from the human GI tract. 2 nt difference to all remaining sequences which were eukaryotic except *Mycoplasma pneumoniae* .	This study	
E. cylindroides 1	52.0		GGTATGAGGAAGGCATCTTCC	*Eubacterium cylindroides* and one unidentified bacteria. 4 nt difference to *Porphyromonas gingivalis* .	This study	There were four 16S ribosomal gene sequences in the database for this strain, but when clustaled only two matched this probe. The E. cylindroides2 probe covers the other two sequences.
E. cylindroides 2	51.0	20	GGTAGAGAGATCGCATGAAC	*Eubacterium cylindroides* . 1 nt difference to eukaryotic sequences and remaining similar sequences had 5 nt divergence or more.	This study	There were four 16S ribsomal gene sequences in the database for this strain, but when clustaled only two matched this probe. The E. cylindroides1 probe covers the other two sequences.
E. cylindroides clust1	53.0	16	CGCGGCATTGCTCGTT	*Clostridium innocuum* , *Eubacterium biforme* , *E. cylindroides* , *E. dolichum* , *E. tortuosum and Streptococcus pleomorphus* . There were also many uncultured bacteria from the gut which should bind to this probe. 1 nt difference to many eukaryotic sequences and no further matches.	Harmsen *et al.* (2002)	The 3' CA was removed to bring the NN temperature further in the range of that required for the microarray.
E. cylindroides clust2	53.0	17	GCGGCATTGCTCGTTCA	*Eubacterium cylindroides* , *Clostridium innocuum* , *Eubacterium biforme* , *Eubacterium tortuosum* , *Eubacterium dolichum* , *Streptococcus pleomorphus* and many other uncultured bacteria from the human GI tract. The next nearest sequence similarity were from microorganisms with 2 nt divergence.	Harmsen *et al.* (2002)	By removing the 5' C from the probe, it brought the NN temperature further in the range of that required for the microarray.

E. formicigenerans	50.0	18	ATGACCGCTTCGTAATGG	*Eubacterium formicigenerans* , *Eubacterium* spp, a Clostridiaceae strain and seven unidentified from human or pig GI tracts. One uncultured bacteria with 1 nt difference and the remaining bacteria had 3 nt differences.	This study	
E. hallii	51.0	17	TTGCACTGCCACCTACG	*Eubacterium hallii* and sixteen *Eubacterium* spp. from the gut. 1 nt difference to eukaryotic sequences and remaining similar sequences had 2 nt divergence or more, with many being uncultured bacteria from the gut	Hold *et al.* (2003)	
Eubacterium hallii , *Clostridium herbivorans* , *Clostridium polysaccharolyticum*	52.0	18	CCAGTTACCGGCTCCACC	*Eubacterium hallii* , *Clostridium herbivorans* , *C. polysaccharolyticum* and nine uncultured bacteria from gut sources. Remaining similar sequences had at least 3 nt difference and were eukaryotic or bacteria unlikely to be found in the gut.	Harmsen *et al.* (2002)	
E. lentum	49.9	16	TCCGCATACTCCTCGC	*Eubacterium lentum* , 1 nt difference to *Neurospora crassa* and a single eukaryotic sequence. No other similar sequences.	This study	
E. limosum	47.0	18	CTCATTGGGTACCGTCAT	*Eubacterium limosum* , *E. callanderi* , *Butyribacterium methylotrophicum* and two uncultured bacteria from extreme environments. 1 nt difference to bacteria from a lake sediment, *Pseudoramibacter* ,an uncultured bacteria from a human subgingival plaque and the pig intestine. 2 nt difference to *Oscillatoria amphigranulata, Eubacterium aggregans* and two uncultured human gut bacteria.	This study	
E. moniliforme	51.0	18	CCGCTAATCCATTTCCCG	*Eubacterium moniliforme* and five uncultured bacteria from the human gut. 2 nt difference to fifteen bacteria including *Actinobacterium* (non-gut source), *Actinomycetes* and *Clostridium neonatale* . 3 nt difference to all remaining sequences.	Schwiertz *et al.* (2002)	
E. multiforme	49.0	18	GTTCCTTCATGCGAAGGT	*Eubacterium multiforme* and five Clostridia from earthworm intestines. 1 nt difference to seven Bacilli from non-gut sources. 2 nt difference to many more Bacilli, again all from non-gut sources. Remaining similar sequences had 3 nt divergence.	Schwiertz *et al.* (2002)	
E. ramulus	49.5	17	ACGACTACTGACGCTGA	*Eubacterium ramulus* and thirteen unidentified bacteria, six of which were from the rumen, human or earthworm GI tracts. 1 nt difference to two unidentified strains and 3 nt difference to all remaining similar hits which were uncultured soil bacteria.	This study	
E. rectale 1	51.0	16	GCTCGGCTTCACAGCT	*Eubacterium rectale* and six uncultured bactreria from the human gut. All remaining similar hits had at least 1 nt difference and were eukaryotic.	Kageyama and Benno (2001)	The ATTT from the 5' end of the primer was removed to bring it into the temperature range required for the microarray.

E. rectale 2	53.0	20	ATTGCTTCTCGGTGCCGT	*Eubacterium rectale* and nine unknown bacteria from the human gut. 2 nt difference to *Zygosaccharomyces bailii.* All remaining similar sequences had at least 3 nt divergence from the probe and were eukaryotic.	Kageyama and Benno (2001)	The 5' and 3' C was removed from the probe to bring the NN temperature nearer to the range required for the microarray.
E. ruminantium 1	47.5	16	CCGAGACTGAGGAAGC	*Eubacterium ruminantium* and remaining identical sequences were all eukaryotic. 1 nt difference or more to many eukaryotic sequences.	This study	
E. ruminantium 2	48.4	21	GCTTCTGAAGAATCATTTGAA	*Eubacterium ruminantium* and eleven unidentified rumen bacteria. 1 nt difference to six other unidentifed rumen bacteria and 3 nt to two more. Remaining similar sequences had 5 nt or more divergence.	This study	
E. siraeum	50.1	19	CTAATGAGACTGCCGTTGA	*Eubacterium siraeum* and twenty-seven uncultured bacteria including from chicken, pig, termite and human GI tracts. 2 nt difference to two uncultured bacteria and eukaryotic sequences. 3 nt difference to many uncultured bacteria.	This study	
E. tortuosum	47.0	20	GGTAACCTACCCATGTAACT	*Eubacterium tortuosum* . The next most similar sequences had 2 and 3 nt different from the probe and all were eukaryotic	Kageyama and Benno (2001)	
E. ventriosum	50.0	16	CGAAGGTGGAGCCGAT	*Eubacterium ventriosum* , *Streptomyces coelicolor* , *Rhodopseudomonas palustris* , *Mesorhizobium loti* and eighteen uncultured bacteria from chicken, rat, goat or landfill sources. 1 nt difference to three uncultured bacteria from the human gut and all remaining similar bacteria were unlikely to be found in the gut.	This study	
Fibrobacter	58.0	17	CCGCGCGTTGGCACGG	*Fibrobacter succinogenes* , *F. intestinalis* , *Mycobacterium tuberculosis* and *M. bovis.* 1 nt difference to *Rhodopseudomonas palustris,* Anopheles gambiae, *Haemaphysalis humerosa and* Pseudomonas synxantha. No other similar sequence matches.	Lin and Stahl (1995) (probeBase)	100% coverage
Firmicutes1[#]	49.0	18	CGGAAGATTCCCTACTGC	Firmicutes (Gram-positive bacteria with low G+C content). Use in eqimolar amounts	Meier *et al.* (1999) (probeBase)	51.2% total coverage.
Firmicutes2[#]	49.0	18	CCGAAGATTCCCTACTGC	Firmicutes (Gram-positive bacteria with low G+C content). Use in eqimolar amounts	Meier *et al.* (1999) (probeBase)	51.2% total coverage.
Firmicutes3[#]	47. Probe base described NN temp as 50°C	18	TGGAAGATTCCCTACTGC	Firmicutes (Gram-positive bacteria with low G+C content). Used in eqimolar amounts	Meier *et al.* (1999) (probeBase)	51.2% total coverage.

F. canadensis 1	50.2	18	TATACAGTCGGCGGCTAA	*Flexibacter canadensis* . 3 nt difference to the only other prokaryote hit; *Clostridum spinosporum* .	This study	The probe covered all strains in the NCBI database whose sequence was of sufficient length.
F. canadensis 2	50.4	18	GTGTAACTGACGCTGAGG	*Flexibacter canadensis* and four uncultured bacteria from non-gut sources. 1 nt difference to over fifty Bacilli and Lactobacilli.	This study	
F. necrophorum	50.1	18	TACGAACGGAGCAGAGAT	*Fusobacterium necrophorum* . 1 nt difference to three eukaryotic sequences. All remaining similar sequences were divergent by 3 nt and were eukaryotic.	This study	The probe covered all strains in the NCBI database whose sequence was of sufficient length.
F. prausnitzii	49.7	16	GGCATCGGGTAGAGGG	*Facealibacterium prausnitzii* , six uncultured *Fusobacterium* spp. from human GI tracts and four further uncultured bacteria from human GI tracts. 1 nt difference to two uncultured bacteria and no other similar sequence matches.	This study	
H. pylori	49.9	20	CAGGATGCTAAATTGGCTAC	*Helicobacter pylori* , 2 nt difference to four other *Helicobacter* spp. and all remaining sequences had at least 4 nt difference to the probe.	This study	
L. acidophilus	48.0	19	GCTGAACCAACAGATTCAC	*Lactobacillus acidophillis* but possibly too specific as only three strains (which have more than 1000 nt of sequence) match the probe out of seven in total. Also two uncultured bacteria from the human GI tract. 1 nt difference to one *L. plantarum* from an unknown source. 2 nt difference to two human gut bacteria and remaining similar sequence matches were divergent by at least 3 nt or more to eukaryotic sequences.	Walter *et al.* (2000)	
Lactobacillales	57.0	24	CACCGCTACACATGRAGTTCCACT	General lactobacilli, streptococci and enterococci probe	This study	
Lactobacilli	50.0	18	GCAAGTCTGATGGAGCAA	General lactobacillus probe; *Lactobacillus paracasei* , *L. suntoryeus* , *L. delbrueckii* , *L. ultunensis* , *L. kalixensis* , *L. salivarius* , *L. gasseri* , *L. casei, L. rhamnosus, L. delbrueckii, L. zeae, L. helveticus, L. crispatus, L. iners, L. saerimmeri, Pediococcus acidilactici* , *P. pentosaceus* and many uncultured bacteria and (probably Lactobacilli). 1 nt difference to three *Lactobacillus* spp. and remaining sequences had 2 nt difference to lactobacilli including *L. plantarum* , *L. fermentum* and *L. helveticus* .	This study	

L. brevis	50.1	17	TCAGCCGTCTAAGGTGG	*Lactobacillus brevis* , *L. perolens* , *L. hetero* , *L. acetotolerans* , *L. spicheri* , *Allisonella histaminiformans* , *Anaerovibrio glycerini* and six uncultured bacteria from equine or rumen sources. 1 nt difference to many other bacteria which included Lactobacilli, Pediococci and unculturables especially from the rumen.	This study	
L. casei [#]	50.0	20	TGCACTGAGATTCGACTTAA	*L. casei* . 4 and 5 nt difference to the next most similar bacterial sequences (Rhizobium).	Ward *et al,* (1999)	
L. crispatus	50.0	20	GTAATGACGTTAGGAAAGCG	*Lactobacillus crispatus* , one *L. jensenii bacteria,* one *L. gasseri* and five unknown from human and chicken sources. 3 nt difference to eukaryotic sequences and two *L. crispatus* species.	Walter *et al,* (2000)	
L. jensenii 1	49.9	20	AGAAGTTCTTCGGAATGGAA	*Lactobacillus jensenii* and *L. psittaci* . 2 nt difference to eukaryotic sequences only. Next similar bacterial sequence match was 3 nt.	This study	
L. jensenii 2	47.0	18	GCTACTTTCGCATGAAAG	*Lactobacillus jensenii* and five uncultured human vaginal *Lactobacillus* spp. 1 nt difference to *Lactobacillus psittaci and* 2 nt to nine strains including *Enterococcus faecalis* and *Eubacterium moniliforme.*	This study	
L. johnsonii [#]	49.0	23	GATGATTTTAGTTCTTGCACTAA	*Lactobacillus johnsonii,* one *Lactobacillus* spp. from yoghurt and 3 unknown bacteria from the pig intestine. 1 nt difference to one uncultured bacterium and remaining similar sequence hits were all eukaryotic. Next nearest bacterial sequence match had 6 nt differences.	Ventura *et al.* (2002)	
L. mucosae	50.1	17	CTGATGGAGCAACACCG	*Lactobacillus mucosae* , *L. antrumi* , *L. gastricus* , *L. vaginalis* , *L. fermentum* , *L. oris* , *L. suebicus* , *L. reuteri* , *L. pontis* , *L. panis* , *L. durianis* , *L. colehominis* , *L. cellobiosus and* many unknown bacteria isolated from the human stomach, mouth and vagina plus chicken, food and the rumen. 1 nt difference to many eukaryotic sequences and 2 nt divergence to further eukaryotes and prokaryotecs unlikely to be present in the gut.	This study	
L. plantarum	49.9	21	AACGAACTCTGGTATTGATTG	*Lactobacillus plantarum* , *L. pentosus* (a close relative to *L. plantarum*) and *L. arizonensis* . 1 nt internal difference to six uncultured bacteria. Remaining bacterial sequences had 4 nt or more difference to this probe.	This study	
L. amylovorus	50.1	17	TGTTGTCAGTTGCCAGC	*L.agilis* , *L. salivarius* , *L. equi* , *L. saerimmeri* and fourteen uncultured bacteria from pig, chicken and horse GI tracts. 2 nt difference to fifteen bacteria including *Lactobacillus* spp.	This study	

L. lactis	48.4	21	TGCTAGAATAGGGAATGATTC	*Leuconostoc lactis* , *L. garlicum* , two *Leuconostoc* spp. from the human gut and one uncultured bacteria from cheese. 1 nt difference to an uncultured bacteria and *L. argentinum.* 2 nt divergence to further *Leuconostoc* spp.	This study	
L. minutus	50.1	17	AGGCGGTCTGTTAGGTC	*Lactobacillus* (*Atopobium*) *minutus* , *A. vaginae* , *A. parvulum* , *A. rimae* , *Pantoea toletana* , *Eubacterium fosser* . Next nearest sequence homology was 2 nt difference and all sequences were eukaryotic.	This study	
L. monocytogenes	47.6	17	ATAAAGTGTGGCGCATG	*Listeria monocytogenes* and one *Listeria* spp. 2 nt difference to all remaining sequences which were eukaryotic.	This study	
M. hypermegale 1	50.0	21	GCAACATTTTGAATAAGGGTG	*Megamonas hypermegale* and one unknown bacteria from the caecum of a chicken. 3 nt difference to *Neurospora crassa* and 4 nt divergence to all remaining bacterial sequences likely to cross hybridise.	This study	
M. hypermegale 2	50.3	18	AGAGATGACCGCATGGAT	*Megamonas hypermegale* and nine uncultured bacteria from the chicken gut. 2 nt difference to three uncultured bacteria. Remaining bacterial sequences had at least 3 nt divergence.	This study	
Methanogens	56 (probeBase described NN temp as 57°C)	22	CTTGTCTCAGGTTCCATCTCCG	*Methanobacterium* spp., *Methanobrevibacter* spp., *Methanosphaera* spp. The probe is very comprehensive. 1 nt difference to *Methanobrevibacter* spp. and remaining similar sequence hits had 3 nt difference to other methanogens.	Raskin *et al.* (1994) (probeBase)	
Methanobacterium spp., *Methanobrevibacter* spp., *Methanosphaera* spp.	57 (ProbeBase described NN temp as 59°C)	22	TACCGTCGTCCACTCCTTCCTC	Specific for *Methanobacterium* spp., *Methanobrevibacter* spp., *Methanosphaera* spp. and many uncultured rumen bacteria. 1 nt difference to ten archeaon cultured from many sources including the human gut. Remaining sequences had at least 3 nt difference to the probe and were mostly eukaryotic.	Raskin *et al.* (1994) (probeBase)	Not ordered for expanded microarray
Methanobrevibacter smithii	50.0	19	ATAGGAGGAAGGAGTGGAC	*Methanobrevibacter smithii* , *M. gottschalkii* , *M. woesei* , *M. thaueri* (all from faeces of humans and animals), twelve unknown bacteria from humans, the rumen contents of a yak and sheep. 2 nt difference to seven eukaryotic sequences. 3 nt difference to many *Methanobrevibacter* spp.	This study	Not ordered for expanded microarray
Methanosphaera stadtmanii	49.5	16	CCTGGCGGAAGCTACT	*Methanosphaera stadtmanii.* Remaining 16S ribosomal gene sequences had 1 nt difference to this probe and all were eukaryotic.	This study	Not ordered for expanded microarray
Nitrospirae	63 (ProbeBase described NN temp as 64°C)	21	CGCCTTCGCCACCGGCCTTCC	Too many 16S ribosomal gene target matches to define full probe specificity using BLASTn	Daims *et al.* (1999) (probeBase)	85% total coverage

M. smegmatis	49.9	16	TCGCATGGCCTGGTAG	*Mycobacterium smegmatis* , *M. flavescens* and *M. marinum* . All remaining similar sequences were eukaryotic.	This study	
O. gallinifaecis 1	49.5	18	CCTATACGACCGAGAGGT	*Ochrobactrum gallinifaecis.* 2 nt difference to *Aquamicrobium defluvium* and 3 nt sequence divergence to many uncultured bacteria.	This study	
O. gallinifaecis 2	43.6	18	ACCAATCACTAGGGAATA	*Ochrobactrum gallinifaecis* . 2 nt or greater difference to all other sequences which were eukaryotic.	This study	
O. formigenes	48.7	19	ACAACCAGTCGAAAGATTG	*Oxalobacter formigenes* and four uncultured bacteria. 2 nt difference to many bacteria.	This study	
P. aerogenes	50.1	18	ATGAGGAAGGCGGTTAGT	*Pasteurella aerogenes* , *Haemophilus paracuniculus* , *Actinobacillus capsulatus* (all from the *Pasteurella* genus) and one uncultured bacteria from the pig gut. 1 nt difference to two eukaryotic sequences. Next most similar bacterial sequence had 3 nt divergence.	This study	
P. pentosaceus	50.2	23	GATTATGACGTACTTGTACTGAT	*Pediococcus pentosaceus* . 4 nt difference to another *P. pentosaceus* . 6 nt divergence to *Staphylococcus epidermidis* .	This study	
P. anaerobius	48.7	17	CGGCATCGTAGATATAT	*Peptostreptococcus anaerobis* , *Peptostreptococcus* spp. and two uncultured bacteria from manure and the pig GI tract. 1 nt difference to eukaryotic sequences and *Shewanella oneidensis* . All bacteria with 2 nt divergence or greater were unlikely to be found in gut.	This study	
P. productus	50.2	17	CAGCGATGTTGAGCGAA	*Ruminococcus (Peptostreptococcus) productus* , *Clostridium coccoides* and 1 unknown *Clostridium* spp.. 1 nt difference to *Bifidobacterium longum* . 2 nt difference to *Salmonella* spp. and *Sinorhizobium meliloti* .	This study	
Acidobacteria1	45 (ProbeBase says 47)	17	GCAGTGGGGAATTGTTC	The Acidobacteria group a phylum and many uncultured bacteria.	Ludwig *et al.* (1997) (probeBase)	44.2% total coverage
Acidobacteria2	54 (ProbeBase says 55)	18	AAGGCGGCATCCTGGACC	The Acidobacteria group a phylum and many uncultured bacteria.	Ludwig *et al.* (1997) (probeBase)	38.4% total coverage
Planctomycetales1	47.0	18	GACTTGCATGCCTAATCC	Planctomycetales	Neef *et al.* (1998) (probeBase)	92.6% total coverage
Planctomycetales2	54.0	19	GCCTTGCGACCATACTCCC	Planctomycetales	Neef *et al.* (1998) (probeBase)	82.7% total coverage
Planctomycetales3	54 (ProbeBase described NN temp as 55°C)	18	GCAGCCACCCGTAGGTGT	Planctomycetales	Daims *et al.* (1999) (probeBase)	75.3% total coverage
P. gingivalis	50.2	16	GTTCAGCCTGCCGTTG	*Porphyromonas gingivalis* and *P. gulae* . 1 nt difference to *Neurospora crassa* and all remaining sequence matches were eukaryotic.	This study	

P. macacae	49.9	19	ACATATCCTGTCACAAGGC	*Porphyromonas macacae* . 1 nt difference to eukaryotic sequences. 4 nt difference to *Saccharomyces cerevisiae* and all remaining similar sequence matches were eukaryotic.	This study	
P. buccalis	49.9	18	CTGTTCCGCCTTTTTGTG	*Prevotella buccalis* . 4 nt difference to the next nearest bacteria sequence match which was *Geobacter sulfurreducens* .	This study	Only example of this strain in the database
P. corporis	49.3	17	CGGCATTAAGTGCTTGC	*Prevotella corporis* and *Prevotella* spp. human oral clone. 2 and 3 nt difference to all remaining similar sequences which were eukaryotic.	This study	Only one example of this strain in the database
P. enoeca 1	49.9	18	ATGTAATCCGAAGCAGGC	*Prevotella enoeca* . 1 nt difference and more to all other sequences which were eukarytoic.	This study	Only one *Prevotella enoeca* 16S ribosomal gene was present in the database
P. enoeca 2	52.3	18	TGACTGACGCTGATGCTC	*Prevotella enoeca* and an uncultured bacteria from the pig gut. 1 nt divergence to *P. buccalis* . Many *Prevotella* spp. and unknown bacteria had 2 nt difference to this probe.	This study	Only one *Prevotella enoeca* 16S ribosomal gene was present in the database
P. ruminicola	42 (53°C annealing temp used by Tajima *et al.*)	18	GGTTATCTTGAGTGAGTT	*Prevotella ruminicola* and thirty uncultured bacteria or *Prevotella* spp. 1 and 2 nt difference to all other sequences which were eukaryotic.	Tajima *et al.* (2001)	
P. granulosum	50.5	18	CTCCTGCTGCATGGTGGG	*Propionibacterium granulosum* , *P. acnes* , *Actinomyces slackii* , *A. israelii* , *Acidithiobacillus thioox* (all Actinomyces group), *Mycobacterium chitae* and many uncultured bacteria from human and rumen sources. 1 nt difference to *A. slackii* , *Streptomyces coelicolor* and two uncultured Actinobacteria. 2 nt difference to an *Actinomyces* spp. oral clone, *Bordetella pertussis* , *Bordetella* spp. and a few other uncultured bacteria.	This study	
P. aeruginosa 1	51.0		GGTAACCGTCCCCCTTGC	*Pseudomonas aeruginosa* , *Cellvibrio mixtus* , *C. fulvus* , *C. vulgaris* (all part of Pseudomonad family), *P. thermotolerans* , *P. anguilliseptica* , *P. citronellolis* , *P. resinovorans* , *P. thermaerum* , *P. alcaligenes* , *P. anguilliseptica* , many uncultured bacteria and one Bacteroidetes spp.. 1 nt difference to three unknowns from non-gut sources and all remaining similar sequence hits had 3 nt divergence or more.	Hogardt *et al.* (2000) (probeBase)	

Pseudomonas general probe	50.0	16	CTTCGGACCTCACGCT	This probe covers a large number of Pseudomonads including *P. aeruginosa*. Also matches sequences from *Teredinibacter turnerae Chryseomonas luteola*, *Lucinoma aequizonata*, *Azotobacter beijerinck*, *Acinetobacter baumannii*, *Rhodococcus erythropolis*, *Flavobacterium lutescens* and many unknown bacteria of which only three came from the equine gut. 1 nt difference to several other bacteria from non-mammal gut sources.	This study	
R. cecicola	50.1	18	CTTAGTAGCCAGCGGTTC	*Roseburia cecicola* and sixteen uncultured bacteria from human, equine and goat GI tracts. Twenty-five microorganisms with 1 nt difference including *Clostridium morbi*, *Ruminococcus schinkii*, *Eubacterium oxidoreducens*, *C. herbivorans* (All from rumen or pig intestine) and the remaining uncultured bacteria were mostly from gut sources. Twenty sequences with 2 nt difference (all uncultured) and remaining sequence similarities had 3 nt divergence or more.		
R. intestinalis 1	51.0	18	TAGTAGCCAGCGGGTAAG	*Roseburia intestinalis* and three uncultured bacteria from the human and pig GI tracts. 1 nt difference to uncultured strains from the termite gut and *Aspergillus parasiticus*. Five unknown bacteria with 2 nt difference isolated from the termite gut and remaining similar sequences were eukaryotic and had 3 nt difference.	Hold *et al.* (2003)	The 3' C of this primer was removed and replaced with a T at the 5' end. This brought down the NN temperature to within the range required for the microarray.
R. intestinalis 2	50.1	16	CCAACCGCAAGGAGGA	*Roseburia intestinalis*, *Peptostreptococcus* spp., *P. anaerobius*, *Leptotrichia* spp., human oral clones and nine uncultured bacteria from equine, rumen and human GI tracts. 1 nt difference to many bacteria, most of which were uncultured from the chicken and human gut.	This study	
R. intestinalis sub-cluster	50.0	18	TTCCAATGCAGTACCGGG	*Roseburia intestinalis*, two *Eubacterium* spp., two *E. rectale* spp., a single *Roseburia* spp., *R. cecicola*, *E. rectale* and *E. ramulus*. 2 nt difference to one uncultured bacteria and 3 nt difference to several other strains which are not found in the gut.	Hold *et al.* (2003)	
R. albus	52.0	19	CCTCCTTGCGGTTAGAACA	*Ruminococcus albus* and two uncultured bacteria from the rumen contents of a yak. 2 nt difference to an uncultured *Eubacterium* spp. from a non-gut source. 3 nt difference to many bacteria including many *Burkholderia* spp., *Nitrosospira* spp., *Acinetobacter johnsonii*, *Peptostreptococcus* spp. and *Clostridium sporosphaeroides*	Wang *et al.* (1997)	

R. albus & *R. flavefaciens* 1	56.0	19	AAAGCCCAGTAAGCCGGCC	*Ruminococcus albus, R. flavefaciens*, thirty uncultured *Ruminococcus* spp. from the human, rat, rumen, pig and chicken GI tracts plus water. 3 nt difference to the next most similar sequences which were all eukaryotic except one *Bacillus* spp. and *Xanthomonas campestris*.	Harmsen *et al.* (2002)	
R. albus & *R. flavefaciens* 2	50.0	20	GAAGTAGAGGTAAGCGGAAT	*Ruminococcus flavefaciens*, *R. albus*, five *Ruminococcus* spp. and twenty-eight unknown bacteria from a human Crohn's disease patient, cow, rumen or pig GI tracts. 1 nt difference to eleven uncultured bacteria from the pig, rat, human and termite intestines. Remaining similar sequences had 3 nt or more difference.	This study	
R. bromii	50.1	23	GATTTCTTCGGAATGAATTTGAT	*Ruminococcus bromii*, two *Ruminococcus* spp., four unknown bacteria from the human gut and three unknown bacteria. 1 nt difference to an uncultured bacteria from the pig intestine and 2 nt divergence to an uncultured bacteria from the rat intestine. Remaining similar sequences had 4 nt differences.	This study	
R. callidus	51.0	20	CGCATAACATCATGGATTCG	*Ruminococcus callidus*, two uncultured *Ruminococcus* spp., from the human gut and two uncultured bacteria from the human gut. 4 nt difference to the next nearest sequences which were all eukaryotic.	Wang *et al.* (1997)	
R. flavefaciens	49.0	20	GGACGATAATGACGGTACTT	*Ruminococcus flavefaciens* and ten uncultured bacteria from gut sources. 1 nt difference to twenty uncultured bacteria including from rumen and waste sites, *Clostridium botulinum* and *R. callidus*. Remaining sequence similarities were divergent by 2 nt or more.	Tajima *et al.* (2001)	
R. lactaris	50.0	19	ACGCAATAAGTAGTCCACC	*Ruminococcus lactaris, R. torques*, *Clostridium scindens*, *C. nexile* and C. fusiformis (all from human gut) plus over fifty uncultured bacteria including *Eubacterium* spp., butyrate-producing bacteria, *Ruminococcus* spp. (all from human gut) and other strains from the human, pig and chicken GI tracts. 1 nt difference (in the middle of the probe) to many uncultured bacteria from gut sources.	This study	
R. obeum#	50.0	20	CTCCTTCTTTGCAGTTAGGT	*Ruminococcus obeum* and thirteen uncultured bacteria from pig, human and soil samples. 3 nt difference to the next most similar bacterial sequence which included *Escherichia coli* and *Shigella flexneri.*	Wang *et al.* (1997)	The 3' T was removed from the probe to bring the NN temperature to within the range of the microarray.

R. torques	50.1	20	GGATGAAGAGGATTGTGACT	*Ruminococcus torques*, an uncultured *Ruminococcus* spp. and an uncultured clone from an elderly human. 1 nt difference to a butyrate producing bacteria from the human gut. 3 nt divergence to all remaining sequences which were eukaryotic.	This study	
Rumin-Eubac-Clost cluster	49-49	19	GCTTCTTAGTCARGTACCG	*Ruminococcus-Eubacterium-Clostridium* cluster	Franks *et al.* (1998)	
Salmonella genus#	49.0	20	TGCGGTTATTAACCACAACA	*Salmonella* general probe. Also specific for *Enterobacter cloacae*, *Leclercia adecarboxylata*, *Citrobacter* spp.. 1 nt difference to approximately twenty other bacteria (*Salmonella* spp., *Citrobacter*, *Enterobacter* spp.or uncultured bacteria). 2 nt difference to many other bacteria including *E. coli* and many uncultured bacteria	Netherwood *et al.* (1999)	
S. enteritidis	50.1	20	CATCCACAGAAGAATCCAGA	*Salmonella enteritidis*, *Salmonellla* Typhimurium LT2 and *Enterobacter agglomerans*. 4 nt difference to the next nearest prokaryotic sequence which was *Enterococcus faecalis*.	This study	
S. maxima	49.0	21	CCGCATAACATTATTGAAAGG	*Sarcina maxima* and an uncultured bacterium from manure wash off. 1 nt difference to *S. ventriculi* and three uncultured bacteria (only one from gut source(a rat)). 4 nt and 5 nt difference to all further sequences which were eukaryotic.	This study	
S. ruminantium 1	49.9	20	AGATGCGAATAGTTTCTTGC	*Selenomonas ruminantium* and twelve other uncultured rumen bacteria. 2 nt difference to two uncultured rumen bacteria. 4 nt difference to all other bacteria.	This study	
S. ruminantium 2	45.0	18	TGCTAATACCGAATGTTG	*Selenomonas ruminatum*, *Mitsuokella multiacida*, *Selenomonas lacticifex*, *Pectinatus frisingensis*, *Pectinatus cerevisiiphilus*, *Anaerovibrio glycerini*, *Anaeroarcus burkinens, Propionispira arboris* and many uncultured rumen and swine bacteria. Fifteen mostly uncultured bacteria with 1 nt difference and all remaining sequences had 2 nt difference or more.	K. Tajima *et al.* (2001)	
S. sphaeroides	49.7	19	GCGTGTAGGTGGATTCTTA	*Sporomusa sphaeroides*, *Sporomusa paucivorans* and a *Sporomusa* spp.. 1 nt difference to two *Sporomusa* spp.. 4 nt difference to any other similar sequences.	This study	
Streptococcus genus	51.0	18	CACTCTCCCCTTCTGCAC	A huge variety of Streptococci. 2 nt difference to all remaining sequences which were eukaryotic.	Trebesius *et al.* (2000) (probeBase)	
S. bovis	49.0	20	CTAATACCGCATAACAGCAT	*Streptococcus bovis*, *S. equinus* (sub-family of *S. bovis*), *S. luteciae* and *S. infantarius*. 1 nt difference to *S. gallinaceus* and two other unknown bacteria. All remaining sequences had at least 2 nt divergence.	Tajima *et al.* (2001)	

S. lactis 1	49.9	20	ACGATGAGTGCTAGATGTAG	*Lactococcus/Streptococcus lactis* , *S. cremoris* and twenty-five unknown bacteria including some isolated from a Crohn's disease patient's bacterial community. 1 nt difference to three strains (*L. lactis* and two unknown bacteria). 2 nt difference to many sequences which included a large number uncultured bacteria (all non-gut bacteria), *Weissella* spp. and *Lactobacillus* spp.	This study	
S.lactis 2	50.1	20	GACATACTCGTGCTATTCCT	*Lactococcus lactis* and ten unidentified prokaryotes which includes some isolated from human faeces and swine pit manure. 1 nt difference to *L. garvieae* (also known as *Enterococcus seriolicida*). 5 nt difference to all remaining prokaryotic sequences.	This study	
S. oralis	50.0	19	AAGAACGAGTGTGAGAGTG	*Streptococcus oralis* , *S. cristatus* , *S. peroris* , *S. parasanguis* , *S. pneumoniae* , *S. mitis* , *S. australis* , *S. salivarius* , over seventy-five unidentified human oral bacteria, *S. vestibularis, S. sanguinis* and many further uncultured bacteria. 3 nt difference to many other *Streptococcus* spp.	This study	
S. thermophilus	50.0	18	CTACAAGATGGACCTGCG	*Streptococcus thermophilus* , *S. salivarius* , *S. vestibularis* , *Lactobacillus casei* , *S. mitis* , and fifty-six uncultured bacteria. 2 nt and 3 nt difference to all remaining similar sequences were eukaryotic.	This study	
T. aceticus 1	50.0	18	GGACGACAACTGACTCTG	*Termitobacter aceticus* . 3 nt difference to *Vibrio cholerae* , *Neurospora crassa* and one eukaryotic sequence.	This study	
T. aceticus 2	50.1	19	TCGCTGATTGTCTTAGTGG	*Termitobacter aceticus* . 2 nt difference to a single prokaryote and remaining bacterial sequence similarities had 3 nt difference or more.	This study	
Thermus thermophilus	50.1	17	TACGCTCAGGCTAGACG	*Thermus thermophilus* , *T. aquaticus* , *T. fiji* , *T. igniterrae* and *T. brockianus* . 1 nt difference to an uncultured *Eubacterium* spp. and 2 nt to all remaining sequences which were *Thermus* spp.	This study	Not ordered for expanded microarray
Thermus thermoph 1[#]	49.0	16	GGTGTGTCCAAAGGGC	*Thermus thermophilus* and *Rhodochaete parvula* . 1 nt difference to eukaryotic sequences only.	This study	
Thermus thermoph 2[#]	49.9	18	GGGAAACTCGGGCTAATC	*Thermus thermophilus* and many other thermophilic bacteria all from extreme environments. 1 nt difference to grassland soil clones and many gammaproteobacteria. 2 nt difference to further uncultured soil bacteria.	This study	
Thermus thermoph 3[#]	51.4	16	CGGGTGAAAGCCTGGG	*Thermus thermophilus* and *T. fiji* . 1 nt difference to *Thermus* spp., *Rhodobacter* spp. or eukaryotic sequences.	This study	

Universal1	54 (ProbeBase described NN temp as 55°C)	18	GCTGCCTCCCGTAGGAGT	Specificity to a huge and diverse range of bacteria.	Amann *et al.* (1990) (probeBase)	90.4% total coverage. Probe ordered for initial preliminary array experiments
Universal2	52.0	18	TTGTACACACCGCCCGTC	Specificity to a huge and diverse range of bacteria.	Seksik *et al.* (2003)	
V. fluvialis	50.4	18	CTACCAAGACCGTGATGC	*Vagococcus fluvialis*, four *Vagococcus* spp. and a single uncultured *Enterococcus* spp. from a chicken GI tract. 2 nt difference to many other *Lactobacillus* spp..	This study	
Veillonella genus	51.0	18	AGACGCAATCCCCTCCTT	*Veillonella dispar*, *V. parvula*, *V. atypica*, *V. caviae*, eighteen uncultured bacteria from the mouth, eight from infant faeces and four bacteria from an unknown source. 1 nt difference to thirteen uncultured bacteria from a lake and five *Veillonella* spp. 3 nt difference to all remaining sequences.	Harmsen *et al.* (2002) (probeBase)	
Verrucomicrobiales	54 (ProbeBase described NN temp as 55°C)	18	GCTGCCACCCGTAGGTGT		Daims *et al.* (1999) (probeBase)	70% total coverage
V. spinosum 1	47.8	16	GAGCAATCCGAGCACT	*Verrucomicrobium spinosum* and one uncultured bacteria from soil. 1 nt difference to a single *Pseudomonas putida* from an unknown source.	This study	
V. spinosum 2	50.1	17	CCTCATGTGGTCGCAAG	*Verrucomicrobium spinosum.* 1 nt difference to three uncultured *Verrucomicrobium* spp.. 2 nt difference from *Bacillus cereus* from an unknown source.	This study	
V. spinosum 3	50.1	18	CAACCTGTCGCTTTCTGA	*Verrucomicrobium spinosum.* 1 nt difference to eukaryotic sequences and no further prokaryotic sequences.	This study	
V. spinosum 4	50.2	17	AGCGAGGAATAGCCCAG	*Verrucomicrobium spinosum*. 2 nt difference to eukaryotic sequences, no further prokaryotic sequences.	This study	
V. spinosum 5	50.9	16	ACCGCAAGGAGGCAAG	*Verrucomicrobium spinosum.* The following organisms whose 16S ribosomal gene sequence matched this probe were all isolated from mud samples: *Desulfovibrio giganteus*, two uncultured *Eubacterium* spp., and three uncultured bacteria from the soil. All other sequences had 3 nt difference to this probe: *Thermus aquaticus*, *Desulfotomaculum reducens and Brucella abortus*.	This study	
W. kandleri 1	44.4	18	TGAAATGAGATGAGAAGC	*Weissella kandleri.* 2 nt difference to one strain of *Lactobacillus kandleri*	This study	
W. kandleri 2	49.9	19	GCTTCAAGTCAAATGCCAA	*Weissella* (*Lactobacillus*) *kandleri*. 3 nt difference to another *L. kandleri.* 4 nt difference to *Bacillus anthracis*.	This study	

Yersinia sp1	51.0	18	GGCAGCGGGAAGTAGTTT	*Yersinia rohdei* , *Y. pestis* , *Y. kristensenii* , *Y. intermedia* , *Y. enterocolitica* , *Y. bercovieri* , *Y. aldovae* , *Y. pseudotuberculosis* , *Y. frederiksenii* and *Y. mollaretii* . 3 nt difference to the next most similar sequence which included *Enterobacter* spp. and *Neisseria gonorrhoeae* . 4 nt difference to further bacterial strains.	Sen (2000)	The 5' C was removed from the beginning of this probe to bring it into temperature range required for the microarray.
Yersinia sp2	54.0	18	CGGCAGCGGGAAGTAGTT	*Yersinia rohdei* and *Yersinia pestis* . 1 nt difference to an *Enterobacter* spp.. 2 nt difference to many other strains but all were isolated from non-gut sources.	Lu *et al.* (2003)	The 5' nucleotide was removed to bring the NN temperature closer to the temperature required range required for the microarray.
Negative control1#	50.0	20	AGTCAGTCAGTCAGTCAGTC	No sequence matches	This study	
Negative control2#	50.0	20	AAGGTTCCAAGGTTCCAAGG	No sequence matches	This study	
Negative control3#	50.0	20	GGAAACCCTTTGGGAAACCC	No sequence matches	This study	
Negative control4#	55.0	20	GGAAACCTGTAGCCCTTTCC	No sequence matches	This study	
Negative control5#	52.0	23	GGGTTTCACAAGGTAAGAATGTG	No sequence matches	This study	
BLON135a-Perfect match probe with 0 nt difference to the *Bifidobacterium longum* 16S ribosomal gene. Probes for melting curves. Note: these probes were not specific. They were used to test at what temp the probes dissociate from the product bound.#	51.0	19	GCCCCATACACCGGAATAG	*Bifidobacterium longum* , *B. infantis* and *B. adolescentis*	This study	Ideally all temperatures should have been the same so comparsion of melting temps with different mis-matches can be undertaken.
BLONG135b-Probe with a 1 nt difference to the *Bifidobacterium longum* 16S ribosomal gene. Probes for melting curves. Note: these probes were not specific. They were used to test at what temp the probes dissociate from the product bound.#	53.0	19	GCCCCATGCACCGGAATAG	*Bifidobacterium animalis*	This study	Ideally all temperatures should have been the same so comparsion of melting temps with different mis-matches can be undertaken.
BLONG135c-Probe with a 2 nt difference difference to the Bifidobacterium longum 16S ribosomal gene. Probes for melting curves. Note: these probes are not specific but they are there just to test at what temp the probes dissociate from the product bound.#	54.0	19	GCCCCAGACTCCGGAATAG	*Bifidobacterium bifidum*	This study	Ideally all temperatures should have been the same so comparsion of melting temps with different miss matches can be undertaken.

* = All probe sequences (5'-3') have a 15 nt dTTP spacer at the 5' end. The 5'-terminal dTTP was aminated to allow chemical coupling to the microarray slide.

# = Probes tested for specificity as described in Table 3.

Pink text indicates probes which fall outside the temperature range of the microarray (48°C-52°C), but were still added to the expanded microarray.

NN refers to the nearest neighbour temperature which describes all the relevant interactions of all the atoms in a DNA duplex structure.

The NN method was used to calculate the predicted melting temperature of a primer according to the sum of the nearest neighbour interactions of all the nucleotides.

Description refers to the predicted bacterial species which will bind to the probe using BLASTn software at the NCBI website.

Nucleotide code: Wobble nucleotides in the probes refer to: Y = C or T; D = A, G or T; R = A or G; K = G or T; W = A or T; S = C or G.
