# Supplementary material for: A short-oligonucleotide microarray that allows improved detection of gastrointestinal tract microbial communities
Source: BMC Microbiol. 2008 Nov 11;8:195. doi: 10.1186/1471-2180-8-195 (PMC2628385; doi:10.1186/1471-2180-8-195)
Supplement: Additional file 5 — Microarray binding levels of the entire 16–21-mer microarray probes from healthy individuals and a UC sufferer. Complete set of normalised data values from three healthy individuals (A, B and C) and an Ulcerative Colitis patient in the active disease and remission states. Entire list of binding levels from the short oligonucleotide probes for healthy individuals (designated A, B, C) and the UC sufferer in the disease and remission states. [file 1471-2180-8-195-S5.pdf]

Additional file 5 - Complete set of normalised data values from three healthy individuals (A, B and C) and an Ulcerative Colitis patient in active disease and remission states

Probe name	Individual A	Individual B	Individual C	Disease	Remission
Acidobacteria1	1.09	0.36	0.50	0.15	0.28
Acidobacteria2	0.05	0.02	0.04	0.02	0.04
Alphaproteobacteria	0.87	0.16	0.22	0.09	0.11
Alpha-Deltaproteobacteria	1.79	0.69	0.27	0.00	0.00

Probe name	Individual A	Individual B	Individual C	Disease	Remission
B.thetaiotaomicron & B.ovatus	12.09	3.88	20.53	0.56	4.08
B.ovatus	0.60	0.17	0.96	0.03	0.28
B.thetaiotaomicron	1.86	0.55	2.52	0.01	0.01
B.vulgatus1	8.10	2.90	3.22	0.67	6.13
B.vulgatus2	0.37	0.36	0.13	0.00	0.03
B.putredinis1	13.01	0.34	0.82	0.38	1.51
B.putredinis2	3.80	0.44	0.38	0.07	0.38
B.putredinis3	0.47	0.06	0.18	0.01	0.09
B.putredinis4	2.45	0.24	0.73	0.13	0.51
B.distasonis1*	0.23	0.18	0.23	0.00	0.02
B.distasonis2*	2.84	0.70	3.41	0.08	1.09
B.fragilis grp*	5.20	1.37	6.46	0.10	1.54
B.alcalophilus	0.00	0.01	0.01	0.00	0.00
B.merdae	1.45	0.09	0.35	0.05	0.49
B.splanchnicus	0.00	0.00	0.00	0.00	0.00
B.stercoris	0.41	0.26	0.12	0.00	0.00

Probe name	Individual A	Individual B	Individual C	Disease	Remission
P.enoeca1	0.00	0.00	0.00	0.00	0.00
P.enoeca2	1.35	0.21	1.09	0.02	0.53
F.canadensis1	0.26	0.06	0.11	0.04	0.17
F.canadensis2	5.19	0.64	1.20	0.56	0.76
Cytophaga-Flavobacterium grp	8.95	1.90	7.17	0.92	2.36
Cytophaga-Bacteroidetes1	16.39	2.84	7.51	0.41	2.84
Cytophaga-Bacteroidetes2	0.00	0.00	0.03	0.00	0.00
Cytophaga-Bacteroidetes3	0.24	0.04	0.12	0.00	0.03
F.candensis1	0.26	0.06	0.11	0.04	0.17
F.candensis2	5.19	0.64	1.20	0.56	0.76

Probe name	Individual A	Individual B	Individual C	Disease	Remission
B.longum grp1*	2.87	0.79	0.42	5.41	2.89
B.longum grp2*	3.18	0.38	0.22	2.99	1.54
B.longum grp3*	7.46	1.51	0.97	9.18	5.17
B.bifidum*	5.20	0.21	0.63	0.15	0.28
B.longum grp & B.breve*	0.10	0.05	0.18	0.00	0.00
B.pseud & catenulatum*	0.03	0.01	0.05	0.08	0.02
B.adolescentis*	0.02	0.00	0.03	0.07	0.11
B.angulatum*	0.00	0.00	0.00	0.00	0.00
B.crossotus	3.64	1.04	0.73	0.02	0.01

Probe name	Individual A	Individual B	Individual C	Disease	Remission
CclusterXIVab*	13.51	3.18	6.03	1.94	1.23
C.coccoides	23.87	6.26	12.60	3.24	1.52
C.clostridiformes1	3.52	0.74	1.18	0.83	1.42
C.clostridiformes2	0.00	0.00	0.00	0.00	0.00

C.symbiosum	0.28	0.22	0.15	0.00	0.00
CclusterIV	1.31	0.34	0.35	0.11	0.07
C.leptum grp*	15.58	5.42	11.09	4.07	3.08
C.leptum 1*	0.05	0.04	0.10	0.21	0.03
C.leptum 2*	0.14	0.10	0.54	0.75	0.19
C.leptum sub-group	4.36	1.66	1.84	1.94	1.00
CclusterI	21.79	3.36	4.84	0.23	0.25
C.butyricium	0.24	0.31	0.11	0.00	0.00
C.nexile	0.53	0.11	0.19	0.10	0.03
C.paraputrificum	0.48	0.04	0.14	0.02	0.02

Probe name	Individual A	Individual B	Individual C	Disease	Remission
CclusterIII	13.09	5.11	2.27	1.27	0.34
C.difficile	4.77	0.73	0.93	0.26	0.33
C.eutactus1	0.05	0.10	0.05	0.00	0.00
C.eutactus2	2.12	1.88	1.29	0.01	0.08
D.piger1	0.00	0.02	0.07	0.00	0.00
D.piger2	0.02	0.01	0.07	0.05	0.16
D.piger3	0.00	0.10	0.04	0.00	0.01
D.piger4	0.02	0.05	0.09	0.41	1.12

Probe name	Individual A	Individual B	Individual C	Disease	Remission
E.coli O157	0.01	0.02	0.08	0.00	0.02
E.coli	0.00	0.00	0.00	0.00	0.00
Enterobacteriaceae1*	3.45	8.39	5.02	1.62	0.29
Enterobacteriaceae2	0.07	0.67	0.46	0.03	0.01
E.rectale1	1.88	0.12	0.51	1.43	0.20
E.rectale2	4.46	0.36	1.00	2.16	0.50
E.hallii	0.83	0.15	0.27	0.07	0.06
EhalliiCherbivoransp	0.00	0.00	0.01	0.01	0.00
E.ventriosum	3.72	1.55	1.55	0.19	0.15
E.formicigenerans	3.39	0.64	0.54	1.10	0.75
E.siraeum	0.86	0.11	0.15	0.20	0.10
E.biforme1	0.09	0.08	0.07	0.23	0.06
E.biforme2	0.15	0.08	0.07	0.13	0.02
E.biforme3	0.26	0.74	0.21	1.74	0.12
E.biforme4	0.42	0.38	0.20	0.65	0.15
E.cylindroides1	2.25	0.37	0.16	0.00	0.01
E.cylindroides2	0.00	0.00	0.00	0.02	0.00
E.cylindroides clust1	12.73	3.14	3.81	0.86	0.17
E.cylindroides clust2	20.44	4.34	4.83	2.66	0.35
F.prausnitzii	0.27	0.37	0.63	0.18	0.25
H.pylori	0.00	0.00	0.02	0.02	0.00

Probe name	Individual A	Individual B	Individual C	Disease	Remission
Firmicutes1*	0.03	0.00	0.01	0.00	0.00
Firmicutes2*	1.37	0.09	0.12	0.25	0.06
Firmicutes3*	0.02	0.00	0.00	0.00	0.00
Enterococcus genus*	0.02	0.00	0.01	0.00	0.00
E.cecorum	0.59	0.11	0.15	0.11	0.13
E.faecium*	0.00	0.00	0.02	0.00	0.00
E.faecalis	0.03	0.01	0.04	0.02	0.02
Lactobacilli	0.33	0.26	0.07	0.01	0.11
L.brevis	0.22	0.11	0.06	0.31	0.32
L.johnsonii*	0.00	0.00	0.01	0.00	0.00
L.casei*	0.00	0.00	0.00	0.00	0.08

L.mucosae	0.07	0.04	0.12	0.01	0.03

**Probe name**	**Individual A**	**Individual B**	**Individual C**	**Disease**	**Remission**
Rumin-Eubac-Clost cluster	7.39	2.50	4.26	1.59	0.80
R.bromii	10.07	1.11	2.98	0.00	0.00
R.lactaris	3.01	0.27	0.33	0.18	0.31
R.torques	1.02	0.01	0.01	0.00	0.00
R.obeum*	0.09	0.03	0.09	0.01	0.08
R.cecicola	1.57	0.87	3.22	0.44	0.20
R.intestinalis1	0.76	0.40	0.57	0.03	0.05
R.intestinalis2	0.10	0.05	0.11	0.00	0.01
R. intestinalis sub-cluster	2.27	0.34	1.42	0.78	0.13
R.albus	0.46	0.13	0.37	0.02	0.03
R.albus & R.flavefaciens1	1.43	0.77	0.38	0.05	0.03
R.albus & R.flavefaciens2	10.63	3.07	1.71	1.22	0.25
R.flavefaciens	19.08	5.61	6.02	11.02	2.32
R.callidus	0.67	0.73	1.22	0.05	0.01
Salmonella genus*	0.00	0.00	0.00	0.01	0.00

**Probe name**	**Individual A**	**Individual B**	**Individual C**	**Disease**	**Remission**
S.oralis	2.92	0.18	0.28	0.27	0.22
S.thermophilus	3.75	0.22	0.19	0.40	0.12
Streptococcus genus	3.15	0.31	0.36	0.92	0.29
S.bovis	0.09	0.05	0.14	0.04	0.04
Verrucomicrobiales	1.60	8.57	7.81	0.01	0.05
T.aceticus1	0.75	0.18	0.18	0.17	0.03
T.aceticus2	0.04	0.00	0.01	0.00	0.00
Veillonella genus	0.85	0.32	0.47	0.36	0.34

**Probe name**	**Individual A**	**Individual B**	**Individual C**	**Disease**	**Remission**
Negative control average 1*	0.05	0.01	0.02	0.03	0.02
Negative control average 2*	0.00	0.00	0.00	0.00	0.00
Negative control average 3*	0.01	0.00	0.01	0.00	0.01
Negative control average 4*	0.00	0.01	0.02	0.00	0.00
Negative control average 5*	0.00	0.00	0.02	0.00	0.00
Thermus thermoph 1*	0.14	0.21	0.38	0.08	0.12
Thermus thermoph 2*	0.84	1.07	1.00	1.06	1.04
Thermus thermoph 3*	1.39	1.12	1.16	1.14	1.16

**Probe name**	**Individual A**	**Individual B**	**Individual C**	**Disease**	**Remission**
Bacteroides*	48.33	9.49	54.52	1.81	12.18
Cytophagales-Bacteroidales grp	61.77	15.34	67.81	2.50	18.64
Cytophaga-Bacteroidetes4	117.52	28.80	84.82	10.89	40.26
Lactobacillales	41.37	21.16	8.95	5.69	5.56
Universal1*	117.54	28.95	46.90	10.97	12.19
Universal2	40.74	15.52	23.85	6.48	5.59

**Probe name**	**Individual A**	**Individual B**	**Individual C**	**Disease**	**Remission**
Negative control average 1*	0.01	0.00	0.00	0.00	0.00
Negative control 1*	0.02	0.01	0.00	0.00	0.00
Negative control 1*	0.07	0.00	0.02	0.03	0.01
Negative control 1*	0.08	0.02	0.04	0.05	0.03
Negative control 1*	0.10	0.03	0.06	0.08	0.06
Negative control 2*	0.00	0.00	0.01	0.00	0.01
Negative control 2*	0.00	0.00	0.02	0.00	0.00
Negative control 2*	0.00	0.00	0.00	0.00	0.00

Negative control 2*	0.00	0.00	0.00	0.00	0.00
Negative control 2*	0.00	0.01	0.00	0.00	0.00
Negative control 3*	0.00	0.00	0.00	0.01	0.00
Negative control 3*	0.00	0.00	0.00	0.00	0.00
Negative control 3*	0.00	0.01	0.00	0.00	0.00
Negative control 3*	0.03	0.00	0.03	0.00	0.01
Negative control 3*	0.00	0.03	0.00	0.02	0.01
Negative control 4*	0.00	0.00	0.01	0.01	0.00
Negative control 4*	0.00	0.01	0.03	0.00	0.00
Negative control 4*	0.00	0.01	0.02	0.00	0.00
Negative control 4*	0.00	0.00	0.00	0.00	0.00
Negative control 4*	0.02	0.01	0.06	0.01	0.00
Negative control 5*	0.00	0.00	0.06	0.00	0.01
Negative control 5*	0.00	0.00	0.00	0.01	0.00
Negative control 5*	0.00	0.00	0.00	0.00	0.00
Negative control 5*	0.00	0.00	0.00	0.00	0.00
Negative control 5*	0.02	0.01	0.05	0.01	0.00

**Probe name**	**Individual A**	**Individual B**	**Individual C**	**Disease**	**Remission**
Thermus thermoph 1*	0.14	0.21	0.38	0.08	0.12
Thermus thermoph 2*	0.75	1.26	1.23	0.91	1.09
Thermus thermoph 2*	0.83	1.06	0.86	0.88	0.82
Thermus thermoph 2*	0.69	0.94	1.13	0.81	0.92
Thermus thermoph 2*	1.17	1.42	1.36	1.41	1.24
Thermus thermoph 2*	1.00	1.31	1.15	1.81	1.65
Thermus thermoph 2*	0.90	1.22	1.00	1.81	1.60
Thermus thermoph 2*	0.87	1.14	0.96	1.06	1.05
Thermus thermoph 2*	0.90	1.17	1.06	1.29	1.20
Thermus thermoph 2*	0.65	0.67	0.67	0.34	0.47
Thermus thermoph 2*	0.58	0.55	0.58	0.31	0.38
Thermus thermoph 3*	0.79	0.72	0.65	0.43	0.53
Thermus thermoph 3*	1.09	0.84	0.81	0.59	0.82
Thermus thermoph 3*	1.30	1.25	1.29	1.30	1.49
Thermus thermoph 3*	1.84	1.53	1.37	1.08	1.12
Thermus thermoph 3*	1.95	1.27	1.63	2.39	1.96
Thermus thermoph 3*	1.69	1.42	1.45	1.74	1.70
Thermus thermoph 3*	1.64	1.30	1.29	1.27	1.29
Thermus thermoph 3*	1.56	1.26	1.26	1.20	1.18
Thermus thermoph 3*	0.66	0.48	0.66	0.28	0.37

* = Probes tested in initial specificity testing

Any probe where a value below 0.09 was recored, was considered as giving no signal.
